# Interview and interrogation methods and their effects on true and false confessions: A systematic review update and extension

**DOI:** 10.1002/cl2.1441

**Published:** 2024-10-10

**Authors:** Mary Catlin, David Wilson, Allison D. Redlich, Talley Bettens, Christian Meissner, Sujeeta Bhatt, Susan Brandon

**Affiliations:** ^1^ Criminology, Law and Society George Mason University Fairfax Virginia USA; ^2^ Department of Psychology Iowa State University Ames Iowa USA; ^3^ Institute for Defense Analyses Alexandria Virginia USA; ^4^ New York New York USA

## Abstract

**Background:**

False confessions are often the product of an interrogation process, and the method by which an interrogation is conducted likely affects both the rate of truthful confessions and false confessions. An optimal interrogation method will maximize the former and minimize the latter.

**Objectives:**

The current study was a partial update and extension of Meissner and colleagues' (2012) prior Campbell systematic review titled *Interview and Interrogation Methods and their Effects on True and False Confessions*. Our objective was to assess the effects of interrogation approach on the rates of true and false confessions for criminal (mock) suspects.

**Search Methods:**

PsycINFO, Criminal Justice Abstracts, and 15 other databases were searched starting October 20, 2022, with the final search conducted on May 23, 2023; together with reference checking, citation searching, and contact with authors to identify additional studies.

**Selection Criteria:**

All eligible studies experimentally manipulated interrogation approach (i.e., accusatorial, information‐gathering, or direct questioning) were conducted with mock suspects accused of wrongdoing where ground truth was known, and included information about confession rates.

**Data Collection and Analysis:**

We used standard methodological procedures expected by The Campbell Collaboration for our selection of studies and data collection. However, we developed our own risk of bias items and analyzed our data using network meta‐analysis methods. Data were synthesized via random‐effects network meta‐analysis based on the logged odds ratio.

**Main Results:**

Across the 27 research articles that provided statistical information sufficient to calculate an effect size, 29 individual studies provided a total of 81 effect sizes. Most studies were conducted with college students in the United States. Overall, our risk of bias assessment indicated that authors generally adhered to double‐blind procedures and avoided selective reporting of outcomes. Of note, however, it was often unclear how violations of the randomization process were dealt with.

For true confessions, there were 12 studies estimating the effect between accusatorial and direct questioning, five estimating the effect between information‐gathering and direct questioning, and another five estimating the effect between accusatorial and information‐gathering. Compared to information‐gathering, on average, the accusatorial conditions observed fewer true confessions, although not statistically significant (combined OR = 0.55, 95% CI 0.29, 1.05). The largest effects were between information‐gathering and direct questioning, with the former producing significantly more true confessions on average (combined OR = 2.43, 95% CI 1.29, 4.59). This model showed good consistency between the direct and indirect effects.

For false confessions, there were 20 studies estimating the effect between accusatorial and direct questioning, 4 studies estimating the effect between information‐gathering and direct questioning, and 7 estimating the effect between accusatorial and information‐gathering. On average, accusatorial conditions yielded more false confessions than direct questioning (combined OR = 3.03, 95% CI 1.83, 5.02) or information‐gathering (combined OR = 4.41, 95% CI 1.77, 10.97), both of which are statistically significant. In contrast, direct questioning and information‐gathering had roughly similar rates of false confessions with nonsignificant and small effects that slightly favored information‐gathering (combined OR = 0.69, 95% CI 0.27, 1.78). This model showed good consistency between the direct and indirect effects.

For true confessions under a six‐node model, most of the direct, indirect, and combined network estimated mean odds ratios were not statistically significant. The only significant effects were for (1) information‐gathering versus direct questioning, with the former resulting in more true confessions (combined OR = 2.57, 95% CI 1.38, 4.78); and (2) accusatorial‐evidence ploy versus information‐gathering with the former resulting in fewer true confessions (combined OR = 0.37, 95% CI 0.16, 0.84).

For false confessions under a six‐node model, we found significant effects for (1) accusatorial‐evidence ploys versus direct questioning, with the former resulting in more false confessions (combined OR = 2.98, 95% CI 1.59, 5.59); (2) accusatorial‐evidence ploys versus information‐gathering, with the former resulting in more false confessions (combined OR = 4.47, 95% CI 1.46, 13.68); (3) accusatorial‐other versus direct questioning, with the former resulting in more false confessions (combined OR = 3.12, 95% CI 1.37, 7.10); (4) accusatorial‐other versus information‐gathering, with the former resulting in more false confessions (combined OR = 4.67, 95% CI 1.61, 13.55); and (5) information‐gathering versus minimization, with the latter resulting in more false confessions (combined OR = 0.25, 95% CI = 0.08, 0.83). No other combined effects were significant. This model should be interpreted cautiously, however, as the *Q* statistics raised concerns regarding model consistency.

**Authors' Conclusions:**

Overall, results support calls for reforming policies related to interviewing and interrogation practices to prohibit the use of accusatorial approaches and require the adoption of approaches that are science‐based.

## 1 PLAIN LANGUAGE SUMMARY

Information‐gathering approaches increase true confessions and decrease false confessions.

### 1.1 The review in brief

Information‐gathering approaches increase the number of true confessions and decrease the number of false confessions when compared to both accusatorial and direct questioning interrogation approaches.

### 1.2 What is this review about?

Ideally, interrogation approaches should maximize the number of true confessions obtained while minimizing the number of false confessions. Problematically, however, some interrogation approaches, such as accusatorial approaches, have been tied to false confessions which have contributed to approximately 12% of known wrongful convictions in the United States. An alternative approach, known as information‐gathering, has considerable evidence showing its utility in reducing false confessions. For the purposes of this review, we compared accusatorial, information‐gathering, and direct question interrogation approaches. This review examined whether interrogation approaches have different effects on the rates of true and false confessions. The review considered evidence regarding the debate about whether the United States should move towards more information‐gathering interrogation approaches.

### 1.3 What is the aim of this review?

This Campbell systematic review examined the effects of accusatorial interrogations on true and false confessions, compared to the effects of information‐gathering and direct questioning interrogations. The review summarizes evidence from 29 high‐quality experimental laboratory studies.

### 1.4 What are the main findings of this review?

#### 1.4.1 What studies were included?

This review included studies that evaluate the effects of interrogation approach on true and false confessions. A total of 27 studies were identified. One article reported three independent studies, thus leading to 29 independent studies included in analyses. The studies span the period from 1996 to 2023 and were conducted largely in the United States.

#### 1.4.2 What interrogation approach produces the most true confessions?

Information‐gathering approaches, on average, produce more true confessions than both accusatorial and direct questioning interrogation approaches. The largest difference is between information‐gathering and direct questioning approaches.

#### 1.4.3 What interrogation approach produces the most false confessions?

Accusatorial approaches, on average, produce more false confessions than both information‐gathering and direct questioning interrogation approaches. The rates of false confessions between information‐gathering and direct questioning interrogation approaches were similar.

### 1.5 What do the findings of this review mean?

Overall, results support calls for practice to continue moving away from accusatorial interrogation approaches. Future research should continue directly comparing accusatorial and information‐gathering approaches; furthermore, future research should consider a more nuanced exploration of accusatorial and information‐gathering tactics and their effect on true and false confessions.

### 1.6 How up‐to‐date is this review?

The review authors searched for studies up to 2023.

## 2 SUMMARY OF FINDINGS

SUMMARY OF FINDINGS (Table 1).

**Table 1:** Summary of findings.

Summary of findings				
Finding	Number of studies	Odds ratio	Certainty	Comments
An accusatorial approach results in more false confessions compared to an information gathering approach	7	4.41 (1.77 to 10.97)	Moderate	Direct, indirect and network effect are highly similar. There is a risk of publication selection bias.
An accusatorial approach results in more false confessions compared to direct questioning	20	3.03 (1.83 to 5.02)	Moderate	Direct, indirect and network effect are highly similar. There is a risk of publication selection bias.
An information gathering approach results in more true confessions compared to direct questioning	5	2.43 (1.29 to 4.59)	Moderate	Number of studies is low. Direct and network effect are statistically significant.
An information gathering approach results in more true confessions compared to accusatory	5	0.55 (0.29 to 1.05)	Low	Effect size is meaningful in size but not statistically significant.

## 3 BACKGROUND

### 3.1 Description of the problem

In 1999, Victoria Bell Banks convinced law enforcement to release her from custody based on a claim that she was not receiving adequate prenatal care while in jail. Later, when the Choctaw County Sheriff came looking for a baby, Ms. Banks claimed she had a miscarriage. The sheriff was suspicious and eventually began questioning Ms. Bank's estranged husband, Medell Banks, about the infant. After several days of intense interrogation, Mr. Banks falsely confessed to the child being born alive and buried near his property. It was not until medical doctors examined Victoria and determined that she was physically incapable of being pregnant that Medell was exonerated, 2 years after being convicted and imprisoned (National Registry of Exonerations & Banks, 2021). It may be surprising that someone would confess to a crime they did not commit (Leo, 2009), let alone a crime that never took place. Researchers, however, have long recognized the problematic nature of false confessions (e.g., Münsterberg, 1908) and false confessions have been identified all around the world (Gudjonsson, 2018), including Europe, Asia, Australia, and New Zealand.

False confessions pose several challenges for the criminal legal system (e.g., Kassin, 2014; Scherr et al., 2020). First, it may be difficult to distinguish between true and false confessions. For example, prisoners were recorded providing one confession to a crime they had committed and to which they had been convicted, and a second confession to a fabricated crime that they had not committed. These records were then presented to college students and police officers who were asked to identify whether the confession they viewed was true or false. While the police officers claimed to be more confident in their judgments about the veracity of the confessions, they were no more accurate than the college students, and both groups performed poorly overall (Kassin et al., 2005).

Second, confessions—whether true or false—tend to persuade juries and judges more than many other pieces of evidence. Mock jury studies have found that confessions are seen as more inculpatory than any other form of evidence (e.g., Kassin & Neumann, 1997) and that even when individuals are informed that a confession was coerced and should legally be disregarded, the confession still influences guilt decisions (Kassin & Sukel, 1997). In fact, though jurors seem able to distinguish between coerced and non‐coerced confessions, they still assign meaningful attributions of guilt when an interrogation produces a confession as both types of confessions are significantly more likely to result in a conviction (Mindthoff et al., 2024).

Finally, research examining forensic confirmation biases (see Kassin et al., 2013) has found that knowing a confession has been obtained can influence forensic experts charged with determining whether fingerprints match (Kukucka & Kassin, 2014) and the conclusions of DNA tests (Dror & Hampikian, 2011). Specifically, information about a confession increases the chances that forensic examiners will determine that a suspect cannot be ruled out or that the suspect is in fact a match to the sample in question. The undue influence of confessions on other evidence creates two problems: (1) legal actors may be unaware that they have been influenced by a (false) confession, and (2) the course of an investigation could be altered by this unintentional strengthening of confirmation biases. Once a suspect confesses and other available evidence is perceived as pointing to the same suspect, it is less likely that an investigator will use their limited resources to continue pursuing other potential leads.

This snowball effect of false confessions is described by the Cumulative Disadvantage Framework (CDF; Scherr et al., 2020). The CDF states that once a false confession occurs, several cognitive processes activate in legal actors and decision‐makers that can culminate in a wrongful conviction. First, when an innocent suspect confesses, law enforcement is less motivated to track down other leads and may turn their attention almost exclusively to the suspect who confessed. Second, that confession can then taint other forensic evidence making the innocent suspect appear even more guilty. Third, if the innocent suspect does not plead guilty, at trial it is unlikely that jurors (and even judges) will be able to ignore the confession evidence once it is introduced, even if instructed to do so. Regardless, in both plea and trial settings, confessions tend to serve as a powerful piece of evidence (see Mindthoff et al., 2024). It comes as no surprise then that individuals end up wrongfully convicted because of a false confession (Scherr et al., 2020). Beyond the harm to the wrongfully convicted individual, wrongful convictions could also mean that the true perpetrator has not been identified and could continue committing crimes. Estimates are that an additional 41,000 crimes per year are committed in the United States by true perpetrators who continued to commit crimes after an innocent individual was targeted for their wrongdoing (Norris et al., 2020). Thus, wrongful conviction creates two wrongs: an innocent individual is punished for a crime they did not commit, and a guilty offender remains unpunished and at large in the community to potentially commit new crimes (see Norris et al., 2020).

The National Registry of Exonerations (NRE) estimates that 12% of the more than 3500 known wrongful convictions in the United States involved a false confession (National Registry of Exonerations, 2020). Of the 123 exonerations recorded across 17 European countries by Eurex, 35% involved a false confession (European Registry of Exonerations). A key driver of wrongful convictions are false confessions, which largely occur during interrogations, and have been shown to be influenced by the interrogation method used. Evaluating the relative performance of the two dominant interrogation methods (i.e., accusatorial and information gathering) can inform police department policies, training regarding interrogation methods, and the broader public debate surrounding police reforms.

### 3.2 Description of the intervention

The method by which an interrogation is conducted likely affects both the rate of truthful and false confessions. An optimal interrogation method will maximize the former and minimize the latter. While there are a variety of methods that could be used to conduct an interrogation, two general styles are an accusatorial approach and an information‐gathering approach (Kelly et al., 2013; Meissner et al., 2015). The current meta‐analysis updated and extended a prior meta‐analysis that focused on accusatorial and information‐gathering approaches to interrogations (Meissner et al., 2012, 2014). Specific techniques associated with each of these approaches are briefly described in the following paragraphs, as they are the most common approaches addressed in the literature (Denault & Talwar, 2023).

The Reid Technique is often referred to as *the* exemplar of the accusatorial approach. As the most common interrogation manual taught to law enforcement in the United States (Kostelnik & Reppucci, 2009), the Reid technique starts with an assumption of guilt and relies on law enforcement's ability to detect deception—typically through nonverbal behavior such as avoiding eye contact. Given the assumption of guilt, the goal of the interrogation is to obtain a confession from the suspect (Inbau et al., 2013), through the use of minimization (e.g., suggestions of leniency, justifications, and rationalizations) and maximization (e.g., refusing to accept denials, exaggerating the severity of the situation, exaggerating and/or fabricating evidence) tactics. In general, minimization and maximization envelope a range of tactics that interrogators can use in an attempt to lessen the suspect's perception of culpability or punishment and increase their perception of the severity of the offense and evidence of guilt, respectively. Both the “soft sell” and “hard sell” approaches of minimization and maximization are intended to elicit a confession from a suspect (Kassin & McNall, 1991). Research has suggested that minimization and maximization can have unique effects on confession rates (e.g., Guyll et al., 2019); therefore we examined the influence of these tactics both together as part of an accusatorial approach and independently. For a more complete discussion of accusatorial tactics, see Kelly et al. (2013).

The PEACE model is an example of an information‐gathering approach that has been adopted by the United Kingdom, Australia, New Zealand, and Norway. The PEACE framework for investigative interviews—regardless of the interviewee's status in the investigation (e.g., witness, suspect, cooperative, noncooperative)—includes Planning and Preparation of the interview, Engaging the interviewee by developing rapport and Explaining the ground rules of the interview process, obtaining a complete Account of the incident under investigation, Closure of the interview, and Evaluation of the interview process (see Bull & Rachlew, 2020; Milne et al., 2008). In the United States, the High‐Value Detainee Interrogation Group (HIG), led by the Federal Bureau of Investigation, has supported the development of science‐based approaches to interrogation that incorporate an information‐gathering framework (see Brandon & Meissner, 2023; Meissner et al., 2017, 2023). The purpose of an information‐gathering interview is to elicit credible information from an interviewee. To that end, PEACE and other information‐gathering models focus on the establishment of rapport and trust between interviewer and interviewee to elicit information, the use of cognitive interviewing tactics (e.g., the Cognitive Interview) to enhance interviewees' memory search, and the strategic presentation of evidence (Strategic Use of Evidence) to challenge the interviewees' account of the crime and evaluate the credibility of the elicited information (see Meissner et al., 2015; Swanner et al., 2016).

We also consider studies that use a control condition, often referred to as direct questioning. Direct questioning, as it is meant to serve as a control comparison in experimental research, typically avoids the use of any particular interrogation tactic(s). Instead, direct questioning involves short, declarative sentences/questions that are goal oriented. For example, asking a participant directly if they committed the act in question or simply asking them to sign a confession statement. In this way, direct questioning is an analog to participants' baseline behaviors. In other words, without the introduction of tactics meant to induce a confession (i.e., accusatorial approaches) or elicit information disclosure (i.e., information‐gathering approaches), direct questioning approaches allow researchers to estimate how often people accused of wrongdoing truthfully or falsely admit guilt.

### 3.3 How the intervention might work

Most confessions are elicited during an interrogation, with researchers suggesting that the interrogation approach could be directly responsible for false confessions (Ofshe & Leo, 1997). Specifically, two factors are believed to influence whether an interrogation will result in a false confession from an innocent person: (1) if interrogators enter an interrogation with an a priori assumption of the suspect's guilt and (2) how rapport‐based versus coercive tactics are used during the interrogation. First, assumptions of guilt can be problematic because interrogators are more likely to use coercive interrogation tactics on those they believe are guilty potentially leading to false confessions (Narchet et al., 2011). Theoretically, then, interrogation approaches that encourage assumptions of guilt will be more likely to induce false confessions, while interrogation approaches that avoid assumptions of guilt should minimize false confessions. Thus, false confessions are a natural consequence of accusatorial interrogations (Mortimer & Shepherd, 1999). In turn, a false confession can influence the interpretation of forensic evidence by investigators, suggesting even more “inculpatory” evidence than existed before the false confession (e.g., Kassin et al., 2013). From there, the chances of innocents accepting a plea deal or being convicted at trial increase dramatically (e.g., Leo, 2009; Appleby & Kassin, 2016). Even if exonerated, the false confession holds severe consequences for exonerees attempting to reenter society (e.g., Kukucka & Evelo, 2019).

In contrast, information‐gathering approaches are argued to reduce false confessions by avoiding presumptions of guilt. Theoretically, by changing the goal of an interview from obtaining a confession to eliciting investigative‐relevant information, these techniques avoid the harmful actions associated with confirmation biases in interrogation settings (e.g., Scherr et al., 2020).

Second, assumptions—or lack thereof—of guilt can influence the use of rapport‐based vs. coercive tactics during interrogations. Rapport, broadly defined, is the interpersonal connection established between the interviewee and the interviewer. Rapport tactics are typically intentional behaviors employed by interviewers to encourage information disclosure from interviewees (see Gabbert et al., 2021). When interviewers use rapport tactics, especially those aimed at aligning the interviewee with the interviewer, interviewees are more likely to perceive rapport. Such increased perceptions of rapport increase the likelihood that interview subjects will decide to cooperate, which in turn increases the amount of information disclosed by the interviewee (Brimbal et al., 2019, 2021; Dianiska et al., 2021). Rapport approaches can be verbal, para‐verbal, or non‐verbal, though a common tactic identified in the literature is to simply engage in active listening. Importantly, information‐gathering approaches recognize that rapport is not a static characteristic and can change over the course of an interaction (Alison & Alison, 2017; Kelly et al., 2016).

In contrast, the Reid Technique only considers rapport as important at the start of the interview phase (Inbau et al., 2013). However, studies show that the use of rapport can, and likely will, wane as more accusatorial approaches are introduced in an effort to obtain a confession (Kelly et al., 2016). Furthermore, by itself, rapport could be used as a form of minimization if the interviewer were to falsely lead interviewees to believe that they were working in their best interest (David et al., 2017; Vallano et al., 2022). Research has shown that by addressing rapport throughout the interview, information‐gathering approaches do, in fact, enhance interviewee cooperation and produce more reliable information (Brimbal et al., 2021; Russano et al., 2024; Vanderhallen & Vervaeke, 2014; Walsh & Bull, 2012). Thus, because information‐gathering approaches both avoid assumptions of guilt and actively build rapport throughout an interrogation, this approach should result in fewer false confessions than accusatorial approaches.

### 3.4 Why it is important to do this review

Meissner and colleagues (2012) conducted a Campbell systematic review and meta‐analysis investigating rates of true and false confessions across information‐gathering, accusatorial, and direct questioning (i.e., control) approaches for both field and experimental (laboratory) studies. The synthesis of the field‐based studies found that both information‐gathering and accusatorial interrogation approaches were more likely to elicit a confession when compared to direct questioning. These findings are limited, however, because field studies cannot establish ground truth (actual guilt or innocence). As such, estimated rates of false versus true confessions are not possible, with the overall rate of obtaining a confession (regardless of its reliability) as the only available metric.

In contrast, the findings from Meissner and colleagues' systematic review of the experimental studies suggested that while both information‐gathering and accusatorial approaches increased the likelihood of true confessions as compared to direct questioning (i.e., control), accusatorial approaches also increased the likelihood of false confessions when contrasted directly with information‐gathering approaches. The authors cautioned against strong claims regarding the comparison of the two approaches because the analysis was based on a small number of studies and they urged future researchers to more explicitly compare information‐gathering and accusatorial approaches (Meissner et al., 2012).

The current review is a partial update of Meissner and colleagues' (2012) review, focused solely on experimental studies to determine the diagnosticity (i.e., the ability to maximize true confessions while minimizing false confessions) of interrogation approaches. Generally speaking, researchers rely on two experimental paradigms to study interrogations: the alt‐key (Kassin & Kiechel, 1996) and the “cheating” (Russano, Meissner, et al., 2005) paradigm. The alt‐key paradigm asks participants to complete a computer task with strict instructions not to touch a specific button on the keyboard that will supposedly cause the computer to crash. At some point in the study, the computer will appear to crash and the innocent participant is then interrogated regarding the allegation of pressing the forbidden alt‐key and asked to sign a confession (Kassin & Kiechel, 1996). The cheating paradigm, on the other hand, involves a confederate working through the study with the research participant. The confederate–participant pair is instructed to complete a series of individual and team problem‐solving questions. The confederate, acting as a member of the research team, will either induce the participant to help them with the individual problem‐solving or not. When induced to assist in the individual problem‐solving task, the participant has broken a rule of the experiment and is effectively guilty of cheating. Here, both innocent and guilty participants are then separated from the confederate and interrogated for the alleged act of cheating before being asked to sign a confession (Russano, Meissner, et al., 2005).

Experimental studies cannot ethically mirror certain aspects of interrogations that happen in the real world and therein have lower external validity than field studies. The tactics and scripts used in experimental studies, however, are based on real‐world interrogation manuals (e.g., Inbau et al., 2013), and are therefore designed to induce meaningful physiological and psychological changes in the accused participants (e.g., Guyll et al., 2019; Normile & Scherr, 2018). As such, experimental studies attempt to model the underlying psychological processes that exist in real interrogations. In addition, to further increase external validity, researchers typically involve some level of deception to maintain psychological realism (see Meissner et al., 2010). Thus, it is reasonable to consider experimental studies as analogous to real‐world interrogations to assess diagnosticity and evaluate the efficacy of interrogative approaches. Such research, thereby, offers evidence‐based considerations for the development of policy related to law enforcement interrogation practices.

To our knowledge, there have been four other meta‐analyses examining interview and interrogation approaches since 2012 (though see Meissner, 2021, for meta‐analyses involving other aspects of investigative interviews). The most recent meta‐analysis examined methodological concerns raised by the “cheating” paradigm (Russano, Meissner, et al., 2005) regarding the treatment of participants who violate random assignment (Redlich et al., 2023). Results suggested that maintaining random assignment through intent‐to‐treat analyses attenuated, but did not eliminate, the effect of innocence on legal decision‐making (i.e., Miranda waiver, confession, guilty plea). The methodological concern informed our assessment of risk‐of‐bias but otherwise is limited in its applicability to the current review as Redlich et al. (2023) did not limit their investigation to studies resulting in confessions. Another recent effort used meta‐analytic approaches to examine the effect of rapport‐building and support tactics on children's disclosure in forensic interviews (Lavoie et al., 2021). The focus on child witnesses, however, limits the applicability of these results to the current update (which focuses solely on juvenile and adult suspects). The third meta‐analysis is more pertinent to our efforts as it examined the prevalence of false confessions across experimental paradigms. Results indicated that the alt‐key paradigm was most likely to result in false confessions regardless of typing speed. Furthermore, compared to all other tactics, false evidence ploys (i.e., lying or bluffing to suspects about evidence) were more likely to result in false confessions (Stewart et al., 2018). The last meta‐analysis examined the social, cognitive, and affective factors associated with true and false confessions obtained using the cheating paradigm (Russano, Meissner, et al., 2005). Results demonstrated that false confessions were associated with perceptions of the consequences of confessing and perceptions of the interrogation context (Houston et al., 2014). None of these meta‐analyses, however, looked at the influence of interrogation approach on suspect true and false confessions. Therefore, updating Meissner and colleagues' (2012) review was important, with particular attention paid to experimental paradigms and different types of accusatorial tactics. Additional lab‐based research conducted since 2010 and advances in the methods of meta‐analysis over the past decade, such as the development of network meta‐analysis and associated software implementations, allowed us to extend prior synthesis work in this area.

## 4 OBJECTIVES

The current study is a partial update and extension of Meissner and colleagues' (2012) Campbell systematic review titled *Interview and Interrogation Methods and their Effects on True and False Confessions*. Our focus in the present meta‐analysis was solely on experimental or laboratory‐based studies. Our objective was to assess the effects of interrogation approach on true and false confession outcomes for juvenile and adult criminal (mock) suspects.

To address our objective, a series of network meta‐analyses were conducted, contrasting accusatorial, information‐gathering, and direct questioning interrogation approaches on their ability to elicit true and false confessions. Like its predecessor, the current meta‐analysis focused on (mock) suspects as the population of interest, interview style as the intervention, and the diagnosticity (i.e., ability to increase true confessions while minimizing the number of false confessions) of the interview styles as the indicator of effectiveness.

To expand on past work, the current effort also examined specific accusatorial tactics (e.g., evidence ploy, minimization) for a more nuanced understanding of the influence of such tactics on true and false confessions. To accomplish this goal, a network meta‐analysis was conducted to compare not only macro‐level interrogation approaches, but also how different techniques within the accusatorial approach (i.e., minimization and maximization) compare to one another and to other interrogation techniques regarding true and false confession rates.

## 5 METHODS

Readers should be aware of a few differences between the protocol and the current review. First, as anticipated, changes were made to the coding protocol (see Supporting Information S6) during the pilot coding protocol.

Changes were made either for clarification or expansion of the information captured. The only information removed from the coding protocol was information about manipulations that were included in the experimental studies beyond the interrogation approach and guilt status of primary interest to this review. However, we include such information in the description provided of each study in the Results section. The coding took place in RedCap instead of LibreOffice as originally anticipated. RedCap, like LibreOffice, allows for the one‐to‐many hierarchy necessary for a meta‐analysis but also simplifies the process of double‐coding.

Second, we originally intended to conduct traditional meta‐analyses as the primary analyses and network‐analyses as secondary. Because network meta‐analysis provides direct effects, indirect effects, and combined effects as output, the initially planned approach seemed redundant. The direct effects duplicate traditional univariate meta‐analytic estimates. Therefore, we focus our analyses on the network meta‐analyses and offer the traditional meta‐analyses.

### 5.1 Criteria for considering studies for this review

All criteria for study inclusion were based on the original review by Meissner and colleagues (2012). However, we have noted where we made departures from those original criteria.

#### 5.1.1 Types of studies

For this update, we included only experimental studies, regardless of publication status, that randomly assigned mock subjects (i.e., not real criminal justice suspects in field studies) to two (or more) interrogation (interview) conditions. The experimental manipulation included the random assignment of an accusatorial or information‐gathering interrogation approach. The two approaches could be compared with each other, compared to a control interrogation technique (e.g., direct questioning). For studies with only accusatorial techniques, we include some contrasts between minimization, maximization, and control tactics. Participants (mock suspects—see below) could be entirely aware of the nature of the study (e.g., some studies challenge participants to “get away” with an act of wrongdoing) or could be deceived to various degrees (e.g., some studies lead participants to believe they are facing academic consequences for the supposed wrongdoing). Any experimental paradigm was eligible (e.g., cheating and alt‐key paradigms) and studies could include more than one manipulated factor; however, manipulated factors not pertinent to our review were not analyzed.

The prior review by Meissner and colleagues (2012) also included field‐based observational studies. These were excluded from this review as our objective focused on the reliability of confessions, which is not possible to assess with field studies (where ground truth remains unknown).

#### 5.1.2 Types of participants

Participants were mock suspects who were accused of some wrongdoing. Studies that included victims or witnesses of wrongdoing exclusively were not eligible. Thus, only data relevant to mock suspects from the included studies were considered for this current analysis. However, the type of mock suspect was not limited by race, age, ethnicity, gender, or any other demographic characteristics.

#### 5.1.3 Types of interventions

For the purposes of this partial update, an interview or interrogation method was defined as (1) an intentional use of one or more (2) established interrogation tactics to (3) induce a confession. An intentional use means that the intervention was part of the experimental manipulations (see Section 5.1.1) where at least part of the interrogation was scripted. In other words, studies where experimenters were allowed complete freedom in how they attempted to get a confession were not eligible for the current review. By established interrogation tactic, we mean those tactics that have been associated with either an accusatorial or information‐gathering approach. For example, false evidence ploys are associated with accusatorial methods and are included in accusatorial interrogation manuals (Inbau et al., 2013). When not identified by name, an accusatorial technique was identified by its goal: to obtain a confession, which is the same goal of the Reid technique (see Inbau et al., 2001). There was only one instance in which the authors had to make this assumption. In this case, the authors described the condition in question as a “guilt bias condition,” which we labeled as accusatorial given that accusatorial approaches, but not information‐gathering approaches, are premised on an assumption of guilt. Accusatorial techniques also include the use of minimization and maximization tactics, which themselves encompass a wide range of more specific tactics (see Kelly et al., 2015, for an overview). Conversely, information‐gathering techniques were identified by their goal to seek investigative‐relevant information, an example being the PEACE model (see Milne & Bull, 1999). Information‐gathering techniques often use cognitive interview and rapport‐building tactics. Any tactic identified by Gabbert et al. (2021) was eligible. There was one instance where a study was coded (by consensus) as containing an information‐gathering condition though it was not explicitly called that by the authors. In this case, the authors had a “rapport” and “no rapport” condition. Given the emphasis of rapport‐building in information‐gathering approaches, we coded the rapport condition as information‐gathering. Direct questioning or control techniques could involve elements of the other two techniques, but the primary approach of control techniques is to use short, declarative sentences/questions that are goal oriented. Finally, to determine that a tactics' purpose was to induce a confession, the tactic must have been introduced before the request for a confession. The temporal ordering of tactic and confession, which was largely described in the description of a study's paradigm or within Section Data Collection and Analysis, 5, was evaluated during the full‐text screening by two independent coders.

#### 5.1.4 Types of outcome measures

##### Primary outcomes

Eligible studies included either true or false (or both) confession rates as the dependent variable. Note that we refer to our primary outcome as confession rates to reflect the language used in the original research, but technically these values are proportions as there is no time element in the denominator. Depending on the study design (e.g., some studies included exclusively innocent participants), the study reported either the number of true confessions (i.e., confessions provided by guilty participants), false confessions (i.e., confessions provided by innocent participants), or both. The ground truth of the confession (i.e., true vs. false confession) was the priority for the coding stage. If a study contained both primary confession and secondary confession information, only the primary confession data was considered. Primary confessions are those provided by the supposed wrongdoer and were the outcome of interest. Secondary confessions are typically defined as either an individual admitting they witnessed an act of wrongdoing, which could make them complicit because of their lack of action (e.g., Swanner et al., 2010), or an individual conveying that the supposed wrongdoer confessed to them (e.g., Wetmore et al., 2014). In either case, the individual confessing was not the supposed wrongdoer and thus was not considered in our analyses.

##### Secondary outcomes

There were no secondary outcomes for this review.

#### 5.1.5 Duration of follow‐up

This was not relevant for the review. The dependent variable was measured during a single laboratory session with the participant.

#### 5.1.6 Types of settings

There was no geographic limitation to study location. However, for pragmatic reasons, only studies published in English were considered.

### 5.2 Search methods for identification of studies

#### 5.2.1 Electronic searches

The listed databases and keywords were heavily influenced by the meta‐analysis being replicated (Meissner et al., 2012). The keyword combinations and filters, however, were generated by the first author of the current effort. When possible, we searched across titles, abstracts, author‐supplied keywords, and indexing terms. All searches were limited to those results available in English. We did not restrict our search or eligibility criteria by publication date, as all relevant studies, even those captured in the original effort, were included in our analyses. The following databases, organized by publisher platforms, were searched:

ProQuest

1. Australia & New Zealand Database
2. Criminal Justice Database
3. ERIC
4. ProQuest Dissertations & Theses Global
5. Psychology Database
6. Social Science Database
7. Sociological Abstracts
8. Sociology Database
9. UK & Ireland Database

EBSCO

1. APA PsycExtra
2. APA PsycInfo
3. Criminal Justice Abstracts
4. National Criminal Justice Reference Services Abstracts
5. Psychology and Behavioral Sciences Collection

Web of Science

1. Conference Proceedings Index: Social Sciences & Humanities
2. Social Science Citation Index

Other

1. CINCH: Australian Criminology Database (Informit)
2. Google (first 10 pages of results)
3. Google Scholar (first 10 pages of results)

The following keywords were used in the systematic search (* denotes that the word could have various endings; e.g., interrogat* could be interrogation or interrogator):

1. Interrogat*
2. Information (gathering)
3. Inquisitorial
4. Interview*
5. Suspect*
6. Confess*
7. “Cognitive interview*”
8. “Conversation management”
9. “Ethical interview*”
10. Disclos*
11. “Strategic evidence”
12. Accusat*
13. “Deception detection”
14. PEACE
15. PACE
16. Adversar*
17. Miranda
18. Coerc*
19. Entrap*
20. “Due process of law”
21. Reid
22. Minimi?*
23. Maximiz*
24. Random*
25. Control
26. Comparison
27. Experiment*
28. RCT
29. Manipulat*
30. Lab*
31. Factorial
32. Guilt*
33. Innocen*
34. Responsib*
35. Commit*
36. Effect

The keywords were condensed to the following search logic for databases that allow for advanced Boolean logic: (interrogat* OR information OR inquisitorial OR interview* OR accusat* OR “deception detection” OR PEACE OR PACE OR adversar* OR REID OR minimi?* OR maximiz* OR “cognitive interview*” OR “conversation management” OR “ethical interview*” OR “strategic evidence” OR miranda OR coerc* OR entrap* OR responsib* OR commit*) AND (random* OR control OR comparison OR experiment* OR RCT OR manipulat* OR lab* OR factorial OR effect) AND (confess* OR disclos*) AND (suspect* OR guilt* OR innocen*).^1^ See Supporting Information for the full searches. Databases were searched starting October 20, 2022, with the final search conducted on May 23, 2023.

#### 5.2.2 Searching other resources

The reference lists for all studies deemed eligible for inclusion were harvested for additional potentially eligible studies. Each eligible study was also used for forward citation searching in Google Scholar. Furthermore, we used several resources to complement the search, including:

1. Meissner, C. A., Redlich, A. D., Bhatt, S., & Brandon, S. (2012). Interview and interrogation methods and their effects on true and false confessions. *Campbell Systematic Reviews*. https://doi.org/10.4073/csr.2012.13 [Lists approximately 80 references]
2. Madon, S., More, C., & Ditchfield, R. (2019). Interrogations and confessions. In N. Brewer & B. Douglass (Eds.) *Psychological science and the law*. New York: Guilford Press. [Lists approximately 75 references]
3. Kassin, S. M., Drizin, S., Grisso, T., Gudjonsson, G., Leo, R. A., & Redlich, A. D. (2010). Police‐induced confessions: Risk factors and recommendations. *Law and Human Behavior, 34*(1), 3‐38. https://doi.org/10.1007/s10979-009-9188-6. [Lists approximately 300 references]
4. Stewart, J. M., Woody, W. D., Pulos, S. (2018). The prevalence of false confessions in experimental laboratory simulations: A meta‐analysis. *Behavioral Sciences & the Law*, *36*(1), 12‐31. https://dor.org/10.1002/bsl.2327. [Lists approximately 100 references]
5. High‐Value Detainee Interrogation Group. (2016). *Interrogation: A review of the Science*. [Lists approximately 780 references]

Finally, the reviewers have many well‐established contacts with researchers studying interviewing and interrogation in the United States and abroad. In Supporting Information S5, we include a list of researchers, both known and unknown, we contacted for unpublished or “in press” studies to possibly include.

### 5.3 Data collection and analysis

#### 5.3.1 Description of methods used in primary research

The typical study randomly assigned mock suspects (volunteers, typically college/university students) to one of two or more experimental conditions representing different interrogation methods. As mentioned previously, the two most common experimental paradigms are the cheating paradigm (Russano, Meissner, et al., 2005) and the alt‐key paradigm (Kassin & Kiechel, 1996). In the cheating paradigm, interrogation methods were typically crossed with guilt status. Guilt status was manipulated by a confederate who either did or did not induce participants to help them solve an independent logic problem. In the alt‐key paradigm, all participants were typically innocent as the computer is programmed to crash regardless of participant action. Thus, participants (randomly) assigned to be innocent can only produce false confessions and participants (randomly) assigned to be guilty can only produce true confessions, allowing true and false confessions rates to be estimated from the same study depending on whether participants were innocent, guilty, or randomly assigned to both guilt conditions. Regardless of guilt, all participants were accused of some wrongdoing. It is possible that the mock suspects could have been entirely aware of the nature of the study (e.g., some studies challenged participants to “get away” with an act of wrongdoing), but mock suspects were typically deceived to various degrees (e.g., some studies led participants to believe they were facing academic consequences for the supposed wrongdoing). At the point of accusation, studies typically manipulated the interrogation method through experimenter scripts before asking for a signed confession from participants (see Stewart et al., 2018, for an overview of typical experimental confession studies). We did not discover any studies with non‐standard designs (e.g., longitudinal).

#### 5.3.2 Selection of studies

Two independent coders screened the titles and abstracts of all studies identified in the digital search for potential eligibility using Abstrackr (Abstrackr.cebm.brown.edu). The inter‐rater agreement (96.92%) was acceptable. The first author trained the coders by going over the pre‐registered protocol, practicing screening a subset together, and then going over a pilot round of abstracts screened independently. Furthermore, the coders met on a weekly basis to discuss any confusion or disagreements as they arose. All studies deemed potentially eligible (i.e., both raters answered “yes” or “maybe” when asked if the study meets all eligibility criteria) were then assessed in their full form against the eligibility criteria. This second round of screening was also conducted by two independent coders who determined final eligibility. The lead author further scanned the reference lists of eligible studies; secondary search sources, such as prior reviews; Google; Google Scholar; and the forward citation searches of eligible studies. Any study identified by the lead author as potentially eligible during this phase was evaluated for eligibility by a second independent coder.

#### 5.3.3 Data extraction and management

All studies deemed eligible for inclusion were coded for key variables (e.g., effect size information) and study characteristics (e.g., publication type) by two independent coders. Discrepancies were resolved through discussion, and when consensus could not be reached, one of the lead reviewers made the final decision.

Coders were trained by the lead reviewers in steps: (1) coders were verbally walked through the code sheet for discussion and clarification, (2) the lead reviewers demonstrated how to code an article in its entirety, and (3) the coders practiced on a small subset of articles for review and feedback from the lead reviewers. This small subset of articles was also used as a testing phase of the initial coding protocol. Any changes made to the coding protocol at this point were incorporated into the previously coded studies. This iterative process continued throughout the coding process with regular meetings to discuss coding issues.

Coders were the first and fourth authors, both of whom had prior experience with meta‐analyses. Generally, coding included four hierarchical data levels: a study level, an experimental condition level, an outcome level, and an effect size level. Using RedCap, we created a database that allowed for the one‐to‐many hierarchical nature of our coding protocol (e.g., one study could include several experimental conditions, measure more than one outcome, and have several effect sizes). Below is a more detailed description of the hierarchical data levels:

- Study level variables included static information (e.g., publication type, publication year, geographic location). As such, there was one record per study at this level of coding.
- Experimental condition‐level coding was conducted for each relevant group of the research design. Thus, there was one record for each eligible experimental condition within a study. For example, if a study included a factor with three levels of interrogation techniques (i.e., accusatorial vs. information‐gathering vs. direct questioning), three condition coding sheets were completed to capture each group. Information specific to each condition or full sample (before or after attrition) was coded at this level, such as sample size and interrogation method, with a variable to indicate at what level of precision the information was reported.
- The outcome level included information specific to each eligible outcome measure. Thus, there was one record per outcome.
- The effect size level included all necessary statistical information to calculate a logged odds ratio (L_OR_) and its variance for each outcome. As such, there was one record per coded effect size. Coders were instructed to identify the most detailed numerical data when coding for effect size information (see Supporting Information S6 for the full coding sheet). When eligible studies did not report all necessary data, we made a good‐faith effort to contact authors to obtain the necessary information.

#### 5.3.4 Assessment of risk of bias in included studies

We assessed the risk of bias in the included studies through a combination of unique coding items we developed specifically for this research literature and items adapted from the Cochrane risk‐of‐bias tool for randomized trials (Higgins, 2019). The tool's language was modified to better fit the characteristics of studies eligible for this review. We excluded items that were not relevant to this literature. The specific items are in the coding protocol (see Supporting Information S6).

More specifically, the risk of bias items addressed the following methodological issues: random assignment to both the interrogation technique and guilt conditions, treatment of violations to the randomization process (e.g., participants assigned to the guilty condition who refused to cheat), attrition, level of deception employed in the study, treatment of participants suspicious of the true purpose of the study, and whether mock interrogators were blind to the guilt status of mock suspects. To address the confession outcome, coders documented any missingness of confession outcomes, including selective reporting of confession outcomes.

#### 5.3.5 Measures of experimental effect

We used the odds ratio as the effect size index. The outcomes were binary, and we were interested in comparing pairs of experimental conditions. Thus, the data could be represented as a 2 × 2 contingency table.

#### 5.3.6 Unit of analysis issues

The unit of analysis was the individual study participant. We encountered no complex issues around the unit‐of‐analysis or unit‐of‐assignment (also the participant) among the eligible designs.

#### 5.3.7 Criteria for determination of independent findings

There were only two eligible outcomes for this review: the rate of true confessions and false confessions. However, because these studies may have any number of experimental conditions, numerous effect sizes may be possible for each outcome. For example, if a study had three conditions (accusatorial, information‐gathering, and direct questioning), there were three possible pairings of these conditions and, as such, three possible effect sizes for each outcome. We coded all possible effect‐size combinations but maintained independence at the analysis stage by performing a network meta‐analysis that takes advantage of the network of comparisons provided by these studies. Cell sizes were collapsed when necessary to create a single effect size to maintain independence (i.e., collapsing across two or more accusatorial conditions). We did not discover any studies that measured outcomes at multiple time points.

#### 5.3.8 Dealing with missing data

We contacted authors to request missing effect‐size data. Any study that met all eligibility criteria but for which we were unable to compute an effect size and were unable to get the needed data from the authors was coded for descriptive information and reported.

#### 5.3.9 Assessment of heterogeneity

We assessed heterogeneity using the *Q*‐test and the *I* ^2^ statistic.

#### 5.3.10 Assessment of reporting biases

As part of our risk‐of‐bias tool, we noted if there was any indication in a coded manuscript that one of the two outcomes of interest was measured but not reported. The greater risk in this literature is publication bias, which we attempted to mitigate by purposefully seeking unpublished work as well as published manuscripts. Publication bias was assessed when there were at least ten effect sizes for a given analysis. We did so in two ways: (1) a visual inspection of the funnel plot and (2) an Egger's regression test. We assessed publication bias using the three‐node networks. Note that these funnel plots center the logged odds ratio around the mean for its contrast pair, enabling an assessment of publication bias for the entire network. That is, both the funnel plot and Egger's regression test are comparison‐adjusted.

#### 5.3.11 Data synthesis

Data were synthesized via random‐effects network meta‐analysis based on the logged odds ratio. The models were estimated using the restricted maximum likelihood (REML) estimator of *τ* ^2^. We assumed a common *τ* ^2^ for the network. A network meta‐analysis extends a traditional meta‐analysis by examining all available comparisons (both direct and indirect) in a network. A network meta‐analysis is particularly suited to our goal as it allowed us to compare the relative effectiveness of interrogation techniques directly. Within this network, we differentiated variations on the interview method, such as maximization and minimization accusatorial tactics. This separation is important to investigate as some studies have found that minimization tactics increase confessions (Guyll et al., 2019; Normile & Scherr, 2018). Others find no differences between direct questioning and minimization tactics (Woestehoff, 2016). Following suggestions from the Campbell methods brief on network meta‐analysis, we present a network diagram, information on inconsistency factors (i.e., tests of heterogeneity both within and between designs), and rankograms of the interrogation approaches (see Wilson et al., 2016). The network meta‐analyses were conducted in R using the *netmeta* package (Rücker et al., 2021).

#### 5.3.12 Subgroup analysis and investigation of heterogeneity

Conducting a moderator analysis within the frequentist statistical framework implemented in the *netmeta* package is currently not possible. It is possible, however, to do so within a Bayesian framework. However, we opted to conduct the moderator analyses at the level of contrast pairs rather than across the network as a whole. We did so because our network is sparse, and our moderator variables do not vary within each node. We conducted moderator analyses on the paradigm type and on the risk of bias items to determine how potential bias could influence the interpretation of our results. Moderator analyses were conducted only when the total number of effect sizes was at least 10 and each cell contained at least 2 effect sizes.

#### 5.3.13 Treatment of qualitative research

Qualitative research was not considered as part of this review.

#### 5.3.14 Summary of findings and assessment of the certainty of the evidence

We provide a Summary of findings Table 1 with the results of the meta‐analyses. However, we did not use GRADE or a GRADE‐like system as we did not believe it appropriate for this review. The focus here was not assessing whether a treatment is effective or ineffective. Rather, we were trying to establish the relative performance of different interview methods in eliciting reliable versus unreliable confessions. The confidence intervals around the mean effect sizes were the first line of information on the certainty of the evidence. The second line of information on the certainty of the evidence was methodological weaknesses identified via our risk of bias assessment. The overall results were interpreted within the context of any weaknesses identified, particularly if they were prevalent across studies.

## 6 RESULTS

### 6.1 Description of studies

Table 2 provides general information on the included studies. Most studies were conducted in the United States (69%) with college students (79%) and used the Russano, Meissner, et al. (2005) cheating paradigm (59%). It is worth noting the variation in the number of attempts made to elicit a confession: some participants were asked once and others were asked more than three times. There also was a lack of information regarding the identity of the individuals conducting the interrogations.

**Table 2:** Description of eligible studies.

Reference	Publication type	Paradigm	Subject	Attempts	Interrogator	Experimental conditions
Blair (2007)	Thesis/Dissertation	Alt‐Key	College Students	2	Unclear	Accusatory Evidence‐Ploy, Accusatory Min/Max, Accusatory Other, Direct Questioning
Cole et al. (2013)	Journal	Other	College Students	3	Unclear	Accusatory Evidence‐Ploy, Direct Questioning
Eastwood et al. (2022)	Journal	Cheating	College Students	3	Unclear	Accusatory Minimization, Information Gathering
Evans et al. (2013)	Journal	Other	College Students	1	Unclear	Accusatory Min/Max, Information Gathering
Guyll et al. (2019)	Journal	Cheating	College Students	3	Unclear	Accusatory Maximization, Accusatory Minimization
Hill et al. (2008)	Journal	Cheating	College Students	Unclear	Unclear	Accusatory Other, Direct Questioning
Huang and Teoh (2019)	Journal	Cheating	College Students	1	Student	Direct Questioning, Information Gathering
Kassin and Kiechel (1996)	Journal	Alt‐Key	College Students	2	Unclear	Accusatory Evidence‐Ploy, Direct Questioning
Klaver et al. (2008)	Journal	Alt‐Key	College Students	1	Unclear	Accusatory Maximization, Accusatory Minimization
Meissner et al. (2011)	Other	Cheating	Unclear	3	Unclear	Accusatory Min/Max, Direct Questioning, Information Gathering
Narchet et al. (2011)	Journal	Cheating	College Students	1	Unclear	Accusatory Other, Direct Questioning
Noc et al. (2023)	Journal	Alt‐Key	College Students	Unclear	Student	Accusatory Min/Max, Information Gathering
Normile and Scherr (2018)	Journal	Cheating	College Students	3	Unclear	Accusatory Evidence‐Ploy, Accusatory Minimization
Normile et al. (2017)	Other	Cheating	College Students	3	Unclear	Accusatory Other, Direct Questioning
Paton et al. (2018)	Journal	Mock Crime	Combination	3	Student	Accusatory Minimization, Information Gathering
Perillo and Kassin (Study 1) (2011)	Journal	Alt‐Key	College Students	2	Unclear	Accusatory Evidence‐Ploy, Direct Questioning
Perillo and Kassin (Study 2) (2011)	Journal	Alt‐Key	College Students	2	Unclear	Accusatory Evidence‐Ploy, Direct Questioning
Perillo and Kassin (Study 3) (2011)	Journal	Cheating	College Students	2	Unclear	Accusatory Evidence‐Ploy, Direct Questioning
Redlich and Goodman (2003)	Journal	Alt‐Key	Combination	2	Unclear	Accusatory Evidence‐Ploy, Direct Questioning
Rigoni (2007)	Thesis/Dissertation	Cheating	College Students	3	Student	Accusatory Other, Direct Questioning, Information Gathering
Russano, Meissner, et al. (2005)	Journal	Cheating	College Students	>3	Unclear	Accusatory Minimization, Direct Questioning
Russano, Narchet, et al. (2005)	Conference Presentation	Cheating	Unclear	Unclear	Unclear	Accusatory Evidence‐Ploy, Direct Questioning
Smalarz et al. (2011)	Conference Presentation	Cheating	Unclear	1	Unclear	Accusatory Evidence‐Ploy, Direct Questioning
Swanner et al. (2010)	Journal	Alt‐Key	College Students	Unclear	Unclear	Accusatory Evidence‐Ploy, Direct Questioning
Villalba (2014)	Thesis/Dissertation	Cheating	College Students	3	Student	Direct Questioning, Information Gathering
Wachi et al. (2018)	Journal	Cheating	Community Members	1	Practitioner	Accusatory Evidence‐Ploy, Direct Questioning, Information Gathering
Wilford and Wells (2018)	Journal	Cheating	College Students	3	Unclear	Accusatory Evidence‐Ploy, Direct Questioning
Woestehoff (2016)	Thesis/Dissertation	Cheating	College Students	3	Unclear	Accusatory Maximization, Accusatory Minimization, Direct Questioning
Wright (2013)	Thesis/Dissertation	Other	College Students	1	Unclear	Accusatory Evidence‐Ploy, Direct Questioning

Confession varied in how it was measured across these studies. Specifically, our search captured studies that measured confession as binary (yes/no); resistance (number of attempts to confession); some combination of compliant (binary), internalized (indication that confessor believed in their own guilt), and confabulated (confessor fabricated details of their wrongdoing); and in terms of information yield. All studies measured confession in a binary fashion, although one study did not report these data. The binary confessed/did not confess is the data used for all effect sizes. We did not analyze the other versions of confession for two reasons: (1) once broken down by interrogation approach, there were too few contrasts to conduct meaningful analyses, and (2) for information yield, it was unclear at what point a suspect could be considered as having confessed. In other words, there is no agreement in the literature regarding whether there is a meaningful difference in guilt judgment between an individual who provides one unit of information and one who provides two.

#### 6.1.1 Results of the search

**Figure 1:**
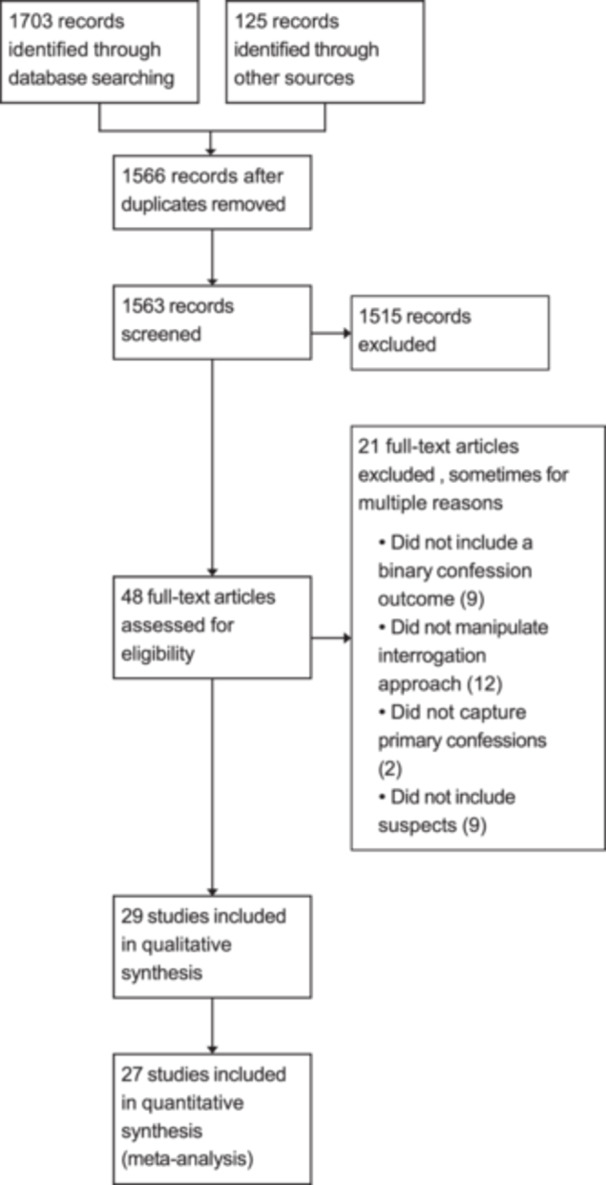
Prisma flow diagram.

Twenty‐seven eligible research articles were identified and coded. Perillo and Kassin (2011) report on the results of three independent studies. As such, 29 independent studies were available for analysis (see Figure 1). The number of experimental conditions varied across these 29 studies, with 16 having 2 conditions, 7 having 3 conditions, 2 having 4 conditions, and 2 having 5 conditions. All studies provided adequate information for analysis or provided the information upon request. Therefore, no studies were excluded for inadequate data. Brief descriptions of the eligible studies and key ineligible studies are provided below. In total, 81 effect sizes were calculated.

#### 6.1.2 Eligible studies

Blair (2007)

This study used the alt‐key paradigm described by Kassin and Kiechel (1996) (see below) with some variations. In the Blair (2007) study, undergraduate student participants were tasked with responding to pictures on a computer and instructed specifically to avoid simultaneously touching the Control, ALT, and Delete keys. Participants were randomly assigned to one of four conditions: presentation or no presentation of false evidence and minimization/maximization or no minimization‐maximization interrogation tactics. In the false evidence condition, the experimenter told the participants that their activity on the computer was monitored by a remote server, which showed that they had pressed the wrong keys. In the minimization/maximization condition, the experimenter stated, “Look, there is no doubt that you pressed the Control, Alt, and Delete keys. That is the only way that this could happen. It has happened a few times during this study. There are usually only two reasons for someone to do something like this. Either they were just goofing around to see what would happen or they were trying to ruin the experiment. I want to believe that you were just goofing around, but the only way I can know it is if you tell the truth and sign this paper. Otherwise, I have to assume that you did it to ruin the experiment.” (p. 178). No differences were found by condition.

Cole et al. (2013)

The purpose of this study was to replicate Kassin and Kiechel (1996) using a different rule violation. Here, participants were accused of breaking a lamp, an act that the authors argued is less ambiguous than hitting an ALT computer key. Fifty‐five undergraduate students were accused of breaking a lamp and randomly assigned to either an incriminating false evidence condition (where a confederate “eyewitness” falsely claimed to have seen the subject break the lamp) or to a no false evidence condition (control). No participants in either condition falsely confessed.

Eastwood et al. (2022)

This study used the cheating paradigm (Russano, Meissner, et al., 2005) described below. Here, 80 participants were either exposed to a dialog‐based or persuasion‐based interrogation before being asked to confess. For our purposes, a dialog‐based approach was considered an information‐gathering interrogation, and a persuasion‐based approach, which used minimization (“Now, I am sure you didn't mean any harm when you shared the answer with the other participant, in fact, I bet you were just trying to be a good person and help the other student out,” p. 3) and justification (“And if asked for help, most people probably would have done the same thing,” p.3) was considered accusatorial. Results indicated no differences in the rate of true confessions; however, the persuasion‐based approach elicited a significantly higher number of false confessions. No innocent participants in the information‐gathering interrogation condition confessed.

Evans et al. (2013)

This study aimed to develop a paradigm based on the cheating paradigm that would allow for gathering empirical data in intelligence‐gathering contexts. Participants were exposed to a rule violation when the confederate brought a cell phone into the room. Innocent participants witnessed the confederate make a phone call during the study, therefore breaking a minor rule, but no further violation occurred. Guilty participants, however, (1) listened to the confederate as they attempted to use an incomplete cheat sheet provided by their roommate; (2) watched as the confederate realized the sheet was incomplete and called their roommate to obtain the remaining answers; and (3) copied all the answers to share with a third friend. Thus, the transgression was complex and provided an opportunity for an assessment of the amount of information yielded from both innocent and guilty participants. In this study, 103 undergraduates were exposed to either an information‐gathering or accusatorial interrogation approach. Participants were more likely to confess when exposed to an information‐gathering interrogation, but there was no effect of culpability (guilt or innocence).

Guyll et al. (2019)

This study measured 296 undergraduates' physiological reactions to being accused of something they either did (i.e., guilty) or did not (i.e., innocent) do. Furthermore, during the interrogation, participants were either exposed to a low‐threat accusation where the violation was described as “pretty minor” and “not a big deal” (p. 311) or a high‐threat accusation where the rule violation was described as “pretty serious” and a “major problem” (p. 311). For the purposes of our study, the “low threat” was considered minimization, and “high threat” was considered maximization. Participants were more likely to confess when they were guilty and exposed to a low‐threat accusation, although the interaction was not significant. The physiological data suggested that the mobilization of resources (i.e., increased physical reactions) aided participants in their resistance to confess, which the researchers suggested explains the difference in confession rates between interrogation approaches.

Hill et al. (2008)

This publication consisted of three separate studies. Only Study 2 was eligible to be included here. Sixty‐four undergraduates self‐selected as either innocent or guilty of cheating (accepting answers from a confederate) during a laboratory task. Half the guilty and half the innocent participants were questioned with guilt‐presumptive questions (accusatorial style), whereas the other half were questioned with neutral questions (control), and confession outcomes were measured. A main effect of interview style did not emerge for either true or false confessions.

Huang and Teoh (2019)

This study aimed to distinguish between the effects of a relationship‐based (where the focus is on a positive interrogator–participant bond) and procedure‐based (where the focus is on interrogation procedure and roles) rapport on true confessions. Using a modified version of the cheating paradigm, all 87 undergraduate students were induced to cheat by a confederate. Only those participants who engaged in the rule‐breaking (*n* = 57) were included in the analyses. These 57 undergraduates were interrogated using either a relationship‐based rapport, procedure‐based rapport, or a direct questioning approach. For our purposes, both types of rapport were considered information‐gathering. Results suggested that how rapport is built matters as those in the procedure‐based rapport‐building condition were more likely to confess than those in the relationship‐based rapport‐building condition.

Kassin and Kiechel (1996)

In this study, college students were invited into the laboratory to participate in a reaction time study. However, the study's actual purpose was to investigate why persons falsely confess. Seventy‐nine participants were placed at a computer and told to type a series of numbers that were read to them, and to not hit the ALT key or the computer would crash. The computer was programmed so that it did crash, and participants were asked one or two times to sign a statement taking responsibility for crashing the computer (i.e., the false confession). Participants were randomly assigned to either hearing the numbers read slowly or quickly and to a false‐evidence or no‐false‐evidence condition. In the false‐evidence condition, a confederate claimed to have seen the participant hit the ALT key. The false evidence condition was considered the accusatorial style, while the no false evidence was considered the control condition. The primary outcome was the number who signed the false confession, ranging from 35% (slow pace, no witness) to 100% (fast pace, witness), depending on the condition.

Klaver et al. (2008)

In this study, 219 college students were exposed to the alt‐key paradigm. Participants were randomly assigned to either a minimization or maximization interrogation condition. In the minimization condition, participants were told, “Don't worry. It was just an accident. You didn't mean to hit the ALT/ESC key. Several participants so far have pressed the ALT/ESC key during this task” (p. 78). In the maximization condition, participants were told, “That file contained all the data collected so far in this study. There is no way to recover the data. It looks like the entire project may be delayed now. Why did you press the ALT/ESC key?” (p. 78). Participants subjected to minimization techniques were more than four times more likely to falsely confess than participants subjected to maximization techniques.

Meissner et al. (2011)

Across two studies, the authors conducted a comparative analysis of information‐gathering and accusatorial interrogation methods. Using the cheating paradigm, guilty and innocent participants were exposed to information‐gathering, accusatorial, or control interrogation tactics. The elicitation of true versus false confessions was recorded. The authors consistently observed that information‐gathering methods reduced the likelihood of false confessions and increased the likelihood of true confessions.

Narchet et al. (2011)

This study investigated the role of interrogators' perceptions of the guilt/innocence of a suspect on the likelihood of eliciting true versus false confessions. Undergraduate students (*n* = 210) participated in a laboratory experiment using the Russano, Meissner, et al. (2005) cheating paradigm. The researchers evaluated the use of various interrogation approaches on the likelihood of confession, including both information‐gathering and accusatorial (minimization and maximization) methods. They found that the accusatorial approaches significantly increased the likelihood of false confessions.

Noc et al. (2023)

Using the alt‐key paradigm, 129 undergraduate students were wrongfully accused of causing a computer to crash by breaking an experimental rule. During a subsequent interrogation, some participants were exposed to the Cognitive Interview for Suspects, an information‐gathering interview, or an accusatorial interview. There was no significant difference in false confession rates between the Cognitive Interview for Suspects and the information‐gathering interview. The accusatory interview, however, elicited significantly more false confessions than the other approaches. For our purposes, both the Cognitive Interview for Suspects and the information‐gathering interview were considered information‐gathering approaches.

Normile and Scherr (2018)

This study assessed resistance to confession and physiological reactivity to true and false accusations of cheating. One hundred and fifty‐four undergraduates were exposed to either minimization or false evidence tactics. Guilty participants were more likely to confess, though there was only a small difference based on interrogation tactics (69.1% in the minimization condition, and 61.6% in the false evidence condition). In terms of physiological reactivity, innocent participants exhibited greater physical response than guilty participants, participants confronted with false evidence reacted more than participants confronted with minimization, and there was a significant interaction such that innocent participants confronted with false evidence evidenced the greatest physiological reactivity.

Normile et al. (2017)

Assessing resistance to confession and physiological reactivity to true and false accusations, 314 undergraduates were exposed to interrogations that varied the order in which participants were exposed to interrogation tactics.

Specifically, participants were either confronted with false evidence, then minimization; minimization, then false evidence; or direct questioning. Guilty participants were significantly more likely to confess compared to innocent participants. Across guilt conditions, confession was most likely when false evidence was introduced first (70%), followed by minimization (63%), and then direct questioning (57%). Differences between confession rates did not differ by order for guilty participants; however, innocent participants interrogated with minimization first or false evidence first were more likely to confess compared to those in the direct questioning condition. Innocent participants in the direct questioning had significantly lower physiological reactivity than innocent participants in the other two conditions.

Paton et al. (2018)

This study investigated the role of interrogation tactic and interview manner on false confessions. One hundred and twenty participants, a combination of students and community members, were falsely accused of stealing a financial gift voucher and were randomly assigned to one of eight experimental conditions. Specifically, the interviewer assumed a friendly or stern demeanor and approached the questioning with minimization, repetitive questioning, leading questions, or nonleading questions. Results indicated that nonleading questions elicited more false confessions when compared to repetitive questions and a friendly interviewer obtained more false confessions than a stern interviewer. No other contrasts were statistically significant. For our purposes, only the non‐leading questions and minimization contrasts were considered.

Perillo and Kassin (2011)

In three studies, the influence of the bluff technique on true (Study 3 only) and false (all studies) confessions was examined. The bluff, categorized as an accusatorial approach here, is when an interrogator insinuates there is incriminating evidence against a suspect. Studies 1 and 2 utilized the alt‐key paradigm. Study 1, which included 79 college students, had five conditions: no‐tactics control, false witness evidence, the bluff technique, false witness and bluff combined, and a witness‐affirmed innocence (another control condition). Relative to the no‐tactic control condition, false evidence and bluff technique conditions increased the rate of false confessions. Study 2 included 44 college students using only the bluff and no‐tactic control conditions. The bluff condition significantly increased false confession rates. Study 3 utilized the Russano, Meissner, et al. (2005) cheating paradigm; 72 participants (college students) were randomly assigned to the guilty or innocent condition. The interview style conditions were bluff versus no‐bluff (control). The bluff condition significantly increased false confessions relative to the no‐bluff control.

Redlich and Goodman (2003)

This study used the Kassin and Kiechel (1996) alt‐key paradigm with some modifications. In addition to college students, juveniles aged 12 and 13, and 15 and 16 years were included to examine if juveniles were more likely to falsely confess than adults. The pace of reading keys was not manipulated and the false evidence was not an eyewitness confederate but rather a fake printout that demonstrated subjects (in that condition) hit the ALT key. Like the original study, the false‐evidence condition was considered accusatorial, and the no‐false‐evidence was considered the control. Also as in the original, all participants were innocent of the mock crime, and thus false confessions were the outcome. Both younger age and false evidence increased the frequency of false confessions.

Rigoni (2007)

Using the cheating paradigm, 180 undergraduates were recruited to participate in a study with a 3 (interrogation approach: inquisitorial vs. accusatorial vs. control) by 2 (guilt: innocent vs. guilty) between‐subjects design. In the full model, guilty participants were significantly more likely to confess than innocent participants. However, there were no differences based on the interrogation approach; nor was there an interaction effect. When the model was collapsed to a single independent variable, interrogation approach, accusatorial approaches were more likely to produce false confessions than both inquisitorial and control approaches. There were no differences in true confessions in the reduced model. Rigoni did find that participants' perceptions of the pressure to confess varied by interrogation approach such that guilty participants exposed to an inquisitorial interrogation felt significantly more pressure to confess than innocent participants exposed to an inquisitorial interrogation.

Russano, Meissner, et al. (2005)

In this cheating paradigm study, 330 undergraduates came to the laboratory to participate in a study on problem‐solving. During this task, half of the participants were induced to cheat via a confederate (the guilty condition), whereas the other half were not (innocent condition). All subjects then were confronted with the possibility of cheating and interviewed using minimization techniques, an explicit offer of leniency, both minimization and an explicit offer (considered accusatorial tactics), or neither (the control condition). The ratio of true to false confessions decreased with the use of accusatorial methods.

Russano, Narchet, et al. (2005)

Using the cheating paradigm, the authors examined the influence of presenting false evidence to guilty and innocent participants on the likelihood of eliciting true versus false confessions, respectively. Participants in the false evidence condition were shown a written confession statement that appeared to have been signed by a second participant (a confederate to the experiment) before being asked to sign their confession statement.

Participants in the no false evidence condition were shown no such statement; they were simply asked to sign their confession statement. Guilty participants were more likely to confess than innocent participants; however, there was no effect of presentation of false evidence on confession rates.

Smalarz et al. (2011)

Using an adaptation of the cheating paradigm, the authors examined the influence of the plausibility of false evidence and guilt on confession rates. Specifically, instead of having a single confederate, roughly a third of participants completed the experiment with two confederates. For these participants, when the interrogator told them there was an eyewitness (i.e., the false evidence), this was plausible. When there was a single confederate, telling the participants there was an eyewitness was implausible. Other participants were not told anything about an eyewitness. All were asked to sign a confession. Guilty participants were more likely to confess. There was also an interaction effect such that guilty participants who were not presented with false evidence were more likely to confess than guilty participants presented with plausible or implausible false evidence.

Swanner et al. (2010)

Across two studies, Swanner et al. used the alt‐key paradigm to investigate secondary confessions (a confession provided by someone other than the suspect). Because secondary confessions were the exclusive focus of Study 2, only Study 1 was included in our analyses. In Study 1, 212 students were randomly assigned to observe or cause a computer crash, that they were accused of causing. Only participants who could provide a primary confession were considered here. Of those who could make a primary confession, half were exposed to false evidence (i.e., an untruthful eyewitness) before being asked to sign a confession, and half were given an incentive (not having to return to the laboratory for a second session). The comparison condition was an interrogation where participants were simply asked to confess. Only when no incentive to sign was provided did false evidence increase the likelihood of false confessions.

Villalba (2014)

One hundred and sixty‐nine undergraduates participated in a cheating paradigm study where they were accused of an act of wrongdoing that they did (i.e., guilty) or did not (i.e., innocent) do. For half of these participants, before they were asked to sign a confession, the interrogator attempted to build rapport with them. The other half were simply asked to sign a confession. For our purposes, the building of rapport was considered an information‐gathering approach. Results indicated that guilty participants were more likely to confess than innocent, but there was no difference based on the building of rapport.

Wachi et al. (2018)

In a modified version of the cheating paradigm, all 234 male participants were asked to cheat by a confederate. Participants who engaged in cheating (*n* = 114) were considered guilty and those who refused to cheat (*n* = 120) were considered innocent. All participants were interrogated by police officers who either approached the interrogation by focusing on the evidence, focusing on building a relationship with the suspect, or on direct questioning. Guilty participants were more likely to confess than innocent, of whom, none falsely confessed. Among the guilty participants, rapport‐building elicited more confessions than direct questioning.

Wilford and Wells (2018)

Using the cheating paradigm, the authors examined the influence of the evidence bluff on guilty and innocent participants' likelihood of giving true versus false confessions, respectively. Importantly, the authors also had a second group of innocent and guilty participants, randomly exposed to an evidence bluff or not, who were asked to accept a guilty plea instead of signing a confession. Only the data from the participants asked to confess were included here. Participants in the evidence bluff condition were told there was camera footage of the incident that had yet to be viewed that could reveal whether cheating had occurred before being asked to sign a confession statement. Participants in the no evidence bluff condition were simply asked to sign a confession statement. Guilty participants were more likely to confess than innocent participants; however, there was no effect of evidence bluff on confession rates.

Woestehoff (2016)

In this two‐study dissertation, Woestehoff tested whether third parties would appreciate the impact of blame mitigation and guilt induction techniques during interrogation (Study 1) in the same way that actors experience the interrogation in practice (Study 2). Only the results from Study 2 were considered here. In Study 2, participants were exposed to the cheating paradigm before being randomly assigned to either a blame mitigation, guilt induction, or direct questioning condition. In the blame mitigation condition (considered minimization in the current analysis), the interrogator attempted to normalize and rationalize the transgression while projecting blame onto the confederate. In the guilt induction condition (considered maximization in the current analysis), the interrogator emphasized the participants' culpability, the suffering caused by the transgression, and made a moral argument about cheating. While guilty participants were more likely to confess, the interrogation approach did not significantly impact confession rates.

Wright (2013)

Across four studies, this dissertation explored how the presentation of false evidence affects its impacts. Specifically, in Study 1, the timing of the presentation of false evidence was manipulated and results suggested that delaying the presentation of false evidence increased its effect. Study 2 expanded Study 1 by including not just an immediate or delayed presentation of false evidence, but an immediate presentation with repeated presentation.

Results from Study 2 found that repetition increased the effect of false evidence. Study 3 looked at the type of false evidence such that participants were randomly assigned to one of four groups: false evidence as a photograph of wrongdoing, false evidence as presented text, both, or neither (i.e., no false evidence; control). Results indicated that participants shown text only were the most likely to confess; there was no difference between false evidence as a photograph and the control group. Only Study 3 met our eligibility criteria and was included in the current analyses. Study 4 manipulated the order in which the different types of false evidence were presented to participants and found that showing false text first was more powerful than photograph evidence.

#### 6.1.3 Ineligible studies

Billings et al. (2007)

Billings et al. examined how reinforcement (i.e., receiving verbal reinforcement that the given answer was correct/desired) influenced children's willingness to falsely confess or express guilty knowledge. Children from kindergarten through 3rd grade watched the staged theft of a toy in their classrooms. Then, children were randomly assigned to one of two interview conditions: control or reinforcement. In the control condition, children were asked straightforward suggestive questions about the theft. In the reinforcement condition, children were asked the same questions but also received reinforcement for the “right” answers. Children in both conditions were also asked if they took the toy (which would be a false confession). In the original meta‐analysis (Meissner et al., 2012), the reinforcement condition was accusatorial (thus, accusatorial vs. control). Billings et al. were excluded from the current endeavor, however, because the children were witnesses of the wrongdoing, not the perpetrators themselves. Therefore, as the children were not suspects, the study was not eligible.

Newring and O'Donohue (2008)

This study utilized a variant of the alt‐key paradigm but was the only study to employ a within‐subject design, which is why it is not included in the current analyses. All subjects were interviewed in a 5‐part process. The first part was a control question of “what happened” (control style). Subsequent parts, which were based on the Reid approach and thus categorized as accusatorial in the original meta‐analysis (Meissner et al., 2012), included requests for written statements, verbal reviews of statements, and explanations of what happened—the latter using the Reid Theme of “reducing the suspect's feeling of guilt by minimizing the moral seriousness of the offense” (p. 93). Twenty‐six undergraduates served as suspects. The main outcome was false confessions.

### 6.2 Risk of bias in included studies

We coded numerous variables that reflected the risk of bias specific to these studies (see Table 3). First, we summarized information related to the randomization of participants to experimental conditions and blinding. Most studies explicitly stated that participants had been randomly assigned to an interrogation approach (86%), and no studies indicated that the interrogation approach was not randomly assigned. A meaningful minority of studies (38%) employed an experimental paradigm that only included guilty or innocent participants. For these studies, random assignment to guilt status was not possible; furthermore, with only a single guilt condition, it was impossible to blind experimenters to the ground truth of participants' guilt. For the remaining studies where random assignment to guilt condition was possible, the majority explicitly stated they had done so (67%). Only a few (17%) did not use random assignment to determine guilt status. Only 48% of studies explicitly stated that experimenters were blinded to guilt status, and 34% of studies either could not (e.g., alt‐key designs) or did not blind experimenters to guilt status. Thus, most studies engaged in random assignment for both interrogation approach and guilt status, providing a reason to be optimistically cautious about our results. However, future researchers should consider blinding experimental manipulations where possible and consider the impact of the lack of blinding on their results.

**Table 3:** Risk of bias.

	Randomization to			Validity threats			
Reference	Interrogation tactic	Guilt status	Blinding	A	B	C	Selective reporting
Blair	Yes	N/A	No	No	Yes	No	No
Cole et al.	Yes	N/A	No	No	Yes	No	No
Eastwood et al.	Unclear	Unclear	Yes	No	Yes	No	No
Evans et al.	Yes	Yes	Yes	No	No	No	No
Guyll et al.	Yes	Yes	Yes	Yes	Yes	No	No
Hill et al.	Unclear	No	Unclear	No	Yes	No	No
Huang and Teoh	Yes	No	Yes	No	No	Yes	No
Kassin and Kiechel	Yes	N/A	No	No	Yes	No	No
Klaver et al.	Yes	N/A	No	No	No	No	No
Meissner et al.	Unclear	Unclear	Unclear	No	No	No	No
Narchet et al.	Yes	Yes	Yes	Yes	No	Yes	No
Noc et al.	Yes	N/A	No	No	No	Yes	No
Normile and Scherr	Yes	Yes	Yes	Yes	Yes	No	No
Normile et al.	Yes	Yes	Yes	Yes	Yes	No	No
Paton et al.	Yes	N/A	No	No	No	No	No
Perillo and Kassin (Study 1)	Yes	N/A	No	No	Yes	No	No
Perillo and Kassin (Study 2)	Yes	N/A	No	No	Yes	No	No
Perillo and Kassin (Study 3)	Yes	Yes	Yes	Yes	Yes	No	No

*Note*: Randomization reflects both whether participants were randomly assigned to an interrogation tactic and whether participants were randomly assigned to a guilt condition. Of note, in the alt‐key paradigm (and other paradigms with only one guilt condition), it was not possible for guilt to be random assignment, so this was coded as N/A. Blinding = whether the mock‐interrogators were aware of participants' guilt statuses. Validity Threats reflects whether the authors considered the following: violations of random assignment (A), participants suspicious about the true nature of the study (B), and differences between mock‐interrogators (C). It should be noted that all three of these threats to validity were coded as to whether or not the authors discussed the possibility, not how they dealt with any potential threats. Scripts = whether the majority of the interrogation was scripted. Selective reporting = whether the authors conceptualized confessions in a way that was not reported.

Second, we focused on potential complications when conducting an experiment, including violations of randomization, suspicion on the part of participants, and any differences between interrogators' confession rates. Across studies, authors typically did not include information about these possible complications in their reports. Specifically, only 38% of researchers addressed violations of random assignment, 55% addressed the possibility that participants were suspicious about the true nature of the study, and 28% addressed differences between interrogators. Importantly, we only speak to whether these particular issues were addressed by authors, not how the authors subsequently dealt with these complications. For example, we cannot speak to how authors determined whether participants were suspicious or not, nor how participants deemed suspicious were subsequently treated. These trends are concerning as they speak directly to questions of internal validity, and without full, transparent reporting, readers cannot effectively judge the rigor of any particular study.

Finally, we summarized information related to selective reporting. Specifically, we looked at how authors conceptualized confession outcomes: binary (confessed vs. did not confess); resistance (i.e., the number of attempts to obtain a confession); compliant, internalized, and confabulated confessions; information yield; and others as defined by the authors. As previously noted, all studies included in the current analyses reported the outcomes of the binary confession outcomes. Coders, however, then confirmed that every other conceptualization of confession was reported such that a reader could interpret the impact of each manipulation on confession as conceptualized. Only one study engaged in selective reporting of confession outcomes.

### 6.3 Effects of interventions synthesis of results

#### 6.3.1 Network meta‐analysis

We estimated four different network meta‐analyses. The first two collapsed all accusatorial conditions into a single condition if multiple such conditions were reported by a single study, generating a network with three nodes: direct questioning (our control condition), information‐gathering, and accusatorial. This network was estimated for our two dependent variables, true and false confessions. The second two network meta‐analyses were conducted keeping the different types of accusatorial conditions separate. We coded five variations across studies: evidence‐ploy, minimization, maximization, minimization and maximization, and other (i.e., any accusatorial style of interrogation that did not fit in one of the aforementioned categories). However, we could not distinguish between minimization/maximization and the other category because most accusatorial interrogation scripts deemed as other were coded as such because they included multiple tactics. Thus, we collapsed these two categories to represent multiple accusatorial tactics, resulting in a network of six nodes: four accusatorial nodes, direct questioning, and information‐gathering. This six‐node network was estimated for both dependent variables, true confessions and false confessions.

##### Three‐node network

###### True confessions

**Figure 2:**
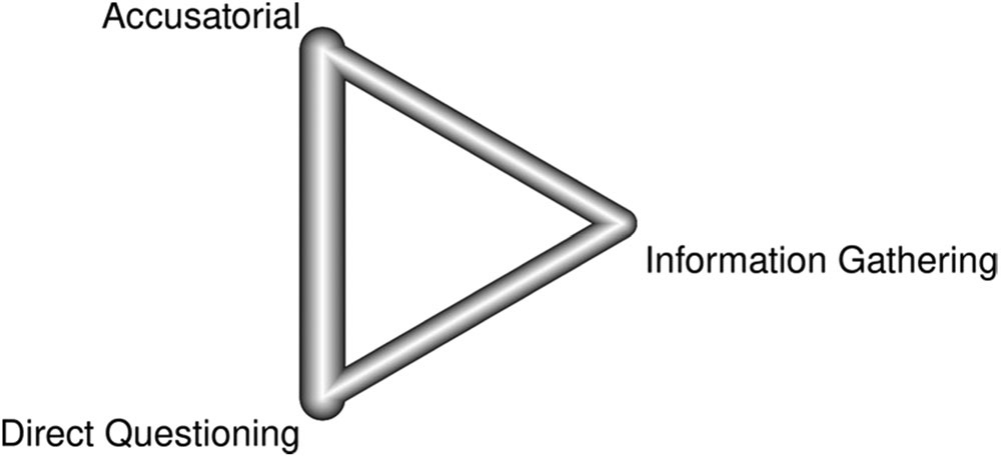
Network graph for three‐node model: True confessions.

**Figure 3:**
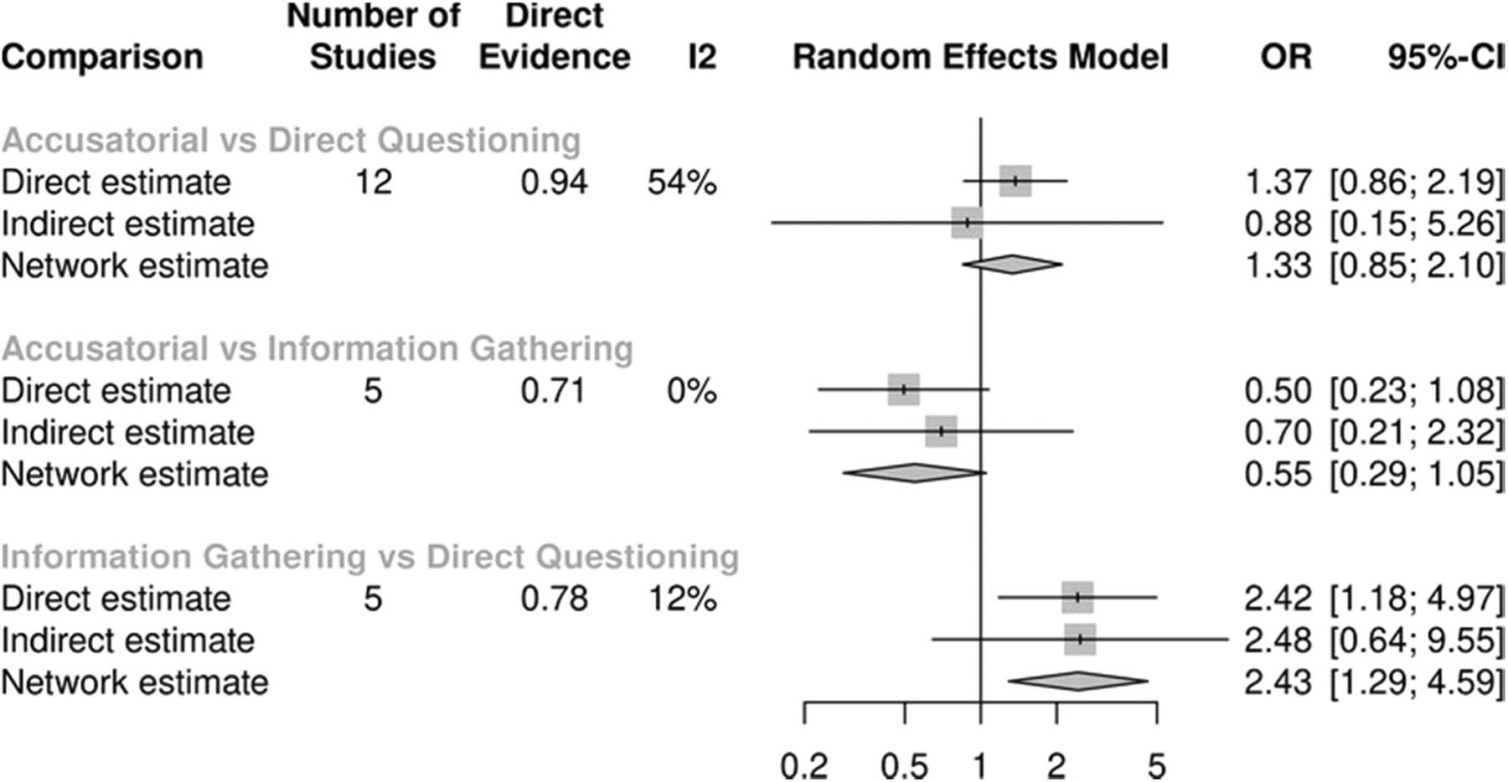
Forest plot for three‐node model: True confessions.

Figure 2 shows the network graphs for the true confession three‐node model. The thickness of the lines (edges) connecting the conditions (nodes) is proportional to the number of studies estimating the effect size (odds ratio) for that paired comparison. There are 12 studies estimating the effect between accusatorial and direct questioning, five estimating the effect between information‐gathering and direct questioning, and another five estimating the effect between accusatorial and information‐gathering. The forest plot (Figure 3) for this network model shows the direct, indirect, and network meta‐analysis effects. These are scaled as odds ratios, and the mean odds ratio and 95% confidence interval are shown graphically and numerically. The “Direct Evidence” column indicates the direct evidence's weight in the combined network estimate.

**Figure 4:**
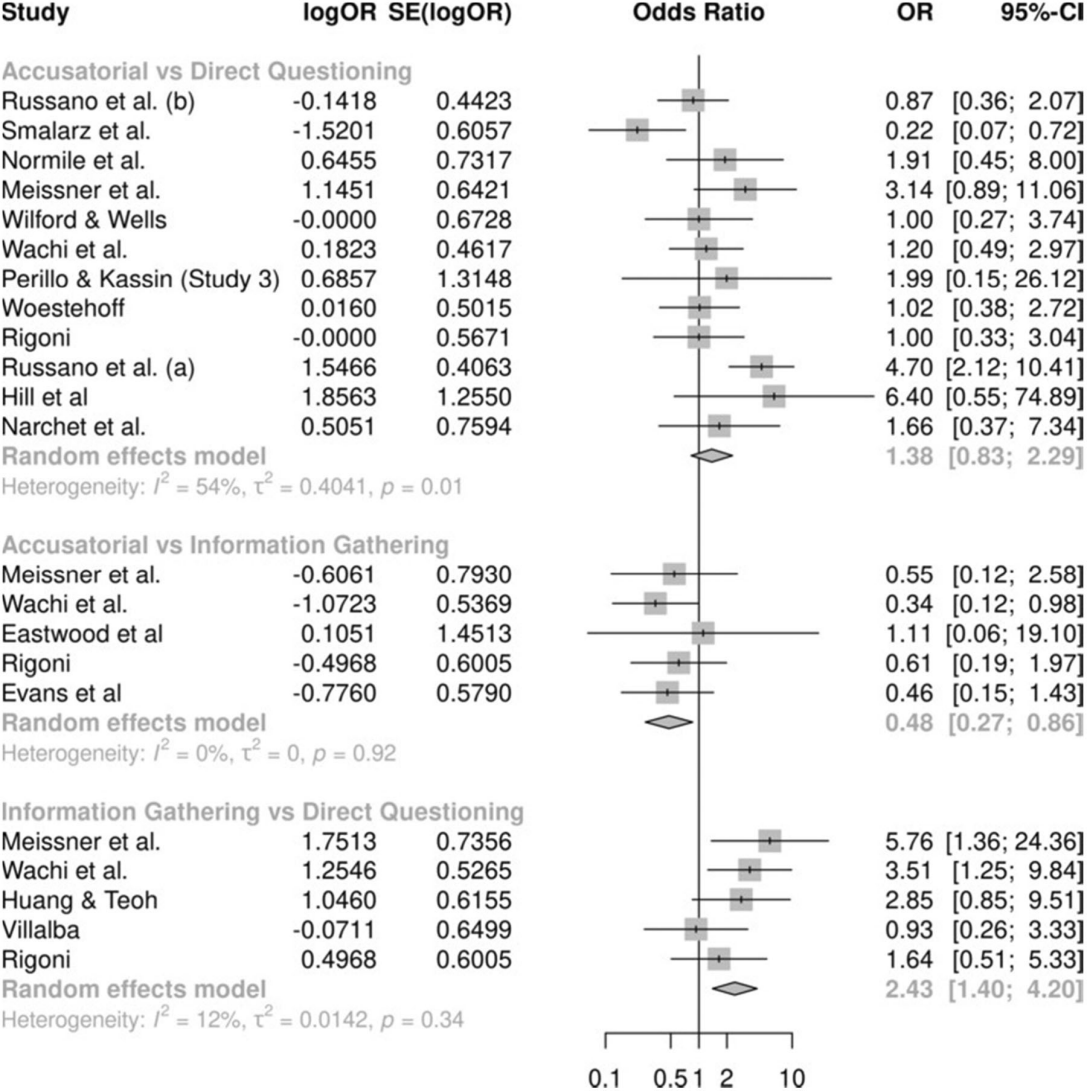
Forest plot of direct evidence for true confessions.

The direct evidence slightly favors accusatorial over direct questions (higher odds ratios indicate more true confessions), although this effect is not statistically significant (OR = 1.37; 95% CI 0.86, 2.19; see Figure 4). The indirect evidence is essentially null (OR = 0.88; 95% CI 0.15, 5.26) with a wide confidence interval. Thus, the indirect evidence contributes only 6% to the combined network estimate that slightly attenuated the direct effect and is also not statistically significant (OR = 1.33, 95% CI 0.85, 2.10).

Compared to information‐gathering, on average, the accusatorial conditions observed fewer true confessions, although the direct, indirect, and combined network effects were not statistically significant (OR = 0.50, 0.70, 0.55, respectively). The largest effects were between information‐gathering and direct questioning, with the former producing more true confessions on average. Although the indirect effects were not significant (OR = 2.48, 95% CI 0.64, 9.55), the direct effects and combined network effect were statistically significant (OR = 2.42, 95% CI 1.18, 4.97; and OR = 2.43, 95% CI 1.29, 4.59, respectively).

The homogeneity and consistency statistics are shown in Table 3. The *Q* within designs assesses the homogeneity of the direct effects within each pairing and is statistically significant, suggesting heterogeneity in the direct effect estimates produced by these studies. The between designs *Q* is not statistically significant, indicating consistency between the direct and indirect estimates, overall. Given the significant *Q* within, we need to interpret the relative rankings shown in Table 4 cautiously.

**Table 4:** Network ranking table for three‐node models.

Outcome	Condition	*p*‐Score
True Confessions	Information Gathering	0.98
	Accusatorial	0.46
	Direct Questioning	0.05
False Confessions	Information Gathering	0.89
	Direct Questioning	0.61
	Accusatorial	0.00

*Note*: Higher odds ratio “good” for true confessions and lower odds ratio “good” for false confessions.

The network ranking, sometimes called a *rankogram*, is useful for assessing the relative rankings for these three conditions on each dependent variable. The ranking order is based on the combined network effect estimate, but the probability (*p*‐score) considers the estimate's precision level. This *p*‐score indicates the certainty, based on the validity of the network model, that each condition is highest ranked (i.e., better than the other conditions). For true confessions, the conditions are ranked *information‐gathering, accusatorial*, and *direct questioning*. The model suggests a high degree of confidence that the information‐gathering approach produces the highest rate of true confessions (*p*‐score = 0.98). The accusatorial approach has slightly less than a 50% chance of having the highest rate of true confessions (*p*‐score = 0.46). There is little chance that direct question produces the most true confessions (*p*‐score = 0.05; see Table 4).

###### False confessions

For false confessions, 20 studies estimate the effect of accusatorial and direct questioning, four studies estimate the effect of information‐gathering and direct questioning, and seven estimate the effect of accusatorial and information‐gathering.

**Figure 5:**
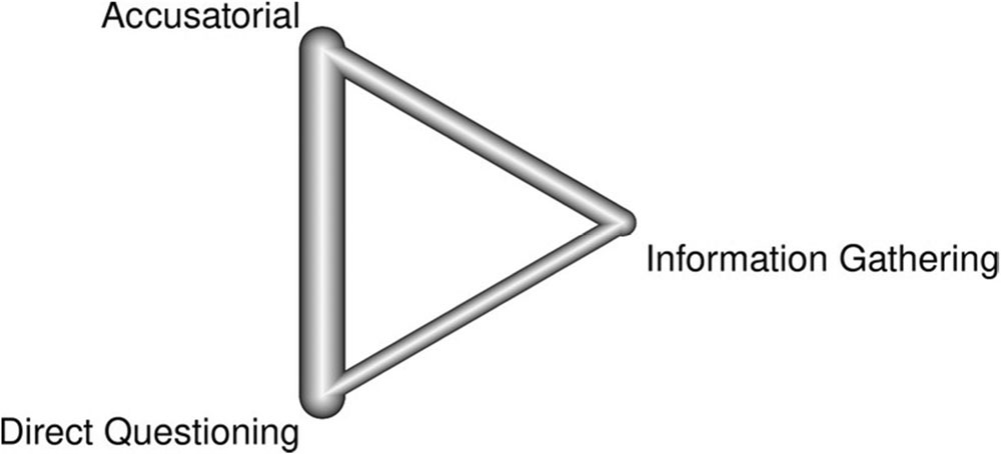
Network graph for three‐node model: False confessions.

**Figure 6:**
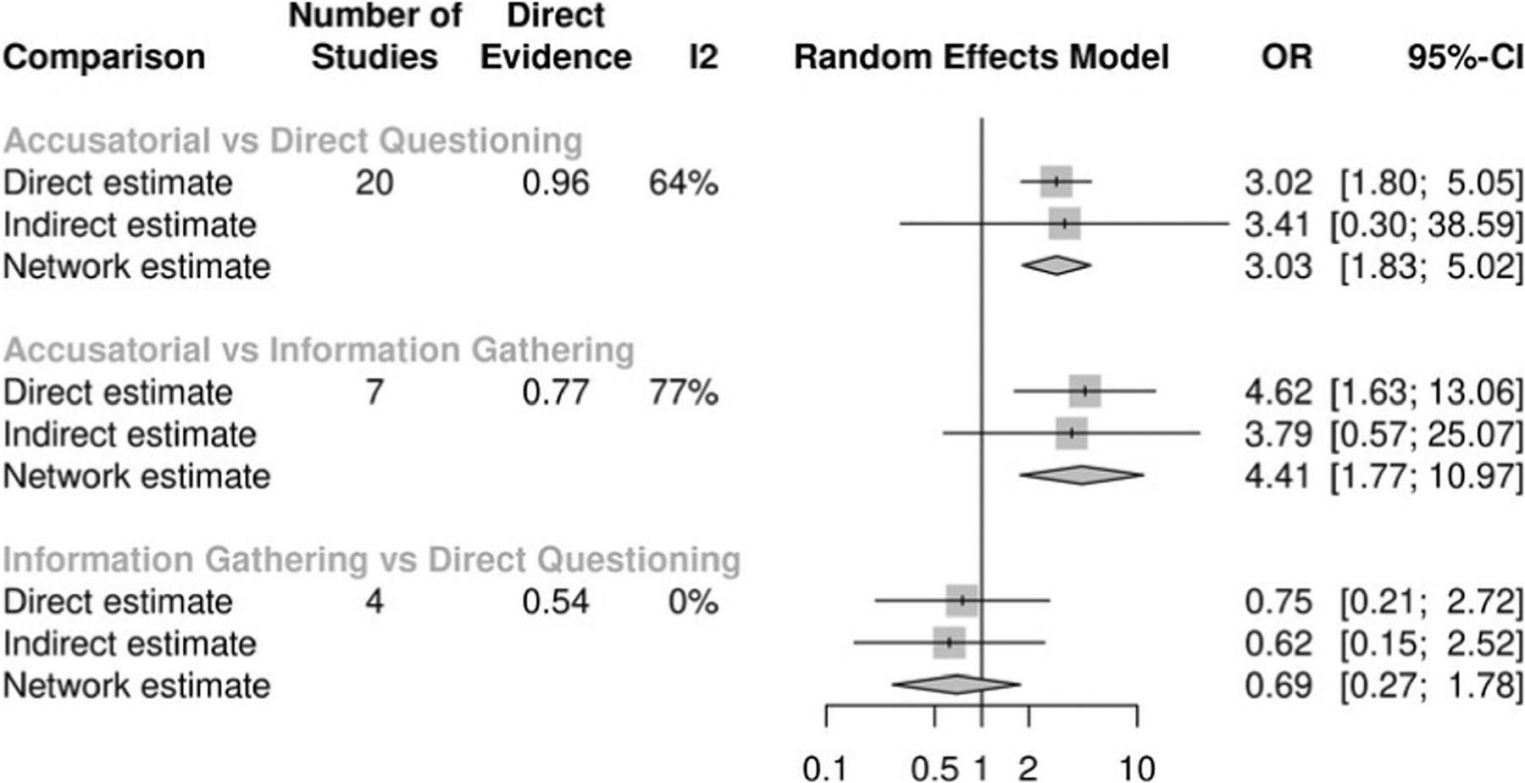
Forest plot for three‐node model: False confessions.

**Figure 7:**
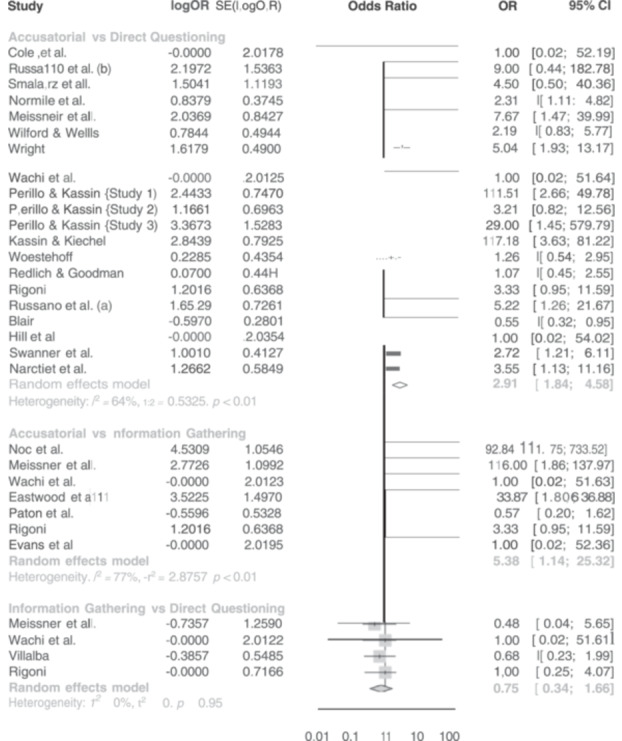
Forest plot of direct evidence for false confessions.

Figure 5 shows the network graph for the false confession three‐node model. Figure 6 shows the forest plot for false confessions for the three‐node model. Notably, the direct and indirect estimates are all highly similar, suggesting a high degree of consistency in this model (*Q* between designs is small and not statistically significant). On average, accusatorial approaches yielded more false confessions than direct questioning or information‐gathering. The direct (see Figure 7) and combined network estimates are statistically significant for both comparisons (direct OR = 3.02 and 4.62, respectively; indirect OR = 3.41 and 3.79; combined OR = 3.03 and 4.41, respectively). These are moderately large effects, with a roughly 180 to 340 percent increase in the odds of a false confession for the accusatorial condition. In contrast, direct questioning and information‐gathering have roughly similar rates of false confessions with nonsignificant and small effects that slightly favor information‐gathering (direct OR = 0.75, 95% CI 0.21, 2.72, indirect OR = 0.62, 95% CI 0.15, 2.52, and combined OR = 0.69, 95% CI 0.27, 1.78). The within designs *Q* is significant for this network with an *I* ^2^ of 67%, indicating substantial heterogeneity in the direct effect estimates.

Compared to true confessions, the ranking changes slightly for false confessions. Note that for false confessions, a low odds ratio is a more desirable outcome (i.e., a low rate of false confessions). Information‐gathering comes out on top (*p*‐score = 0.89) but is followed by direct questioning (*p*‐score = 0.61). Thus, we have insufficient evidence to conclude that information‐gathering reduces false confessions relative to direct questioning (the latter has roughly a 3/5th's chance of being the best); the evidence slightly favors information‐gathering, which has roughly a 9/10th's chance of being the best). We have strong evidence that the accusatorial approach is inferior at minimizing false confessions (*p*‐score < 0.001; see Table 4).

##### Six‐node network

###### True confessions

**Figure 8:**
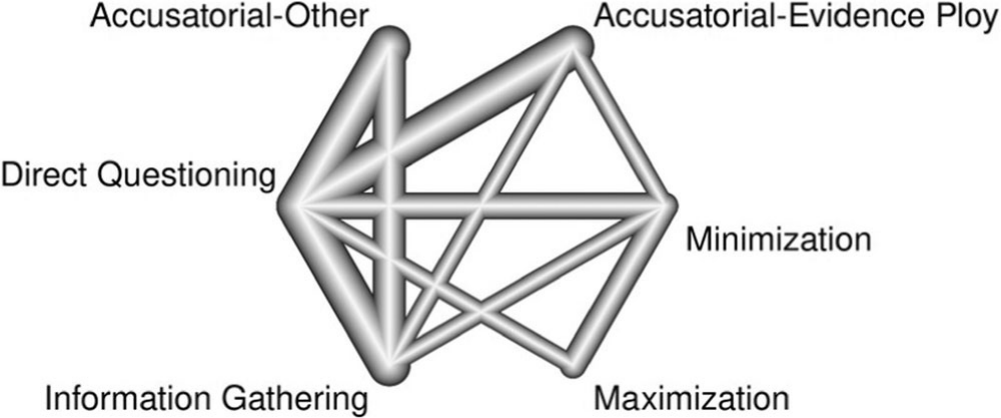
Network graph for six‐node model: True confessions.

**Figure 9:**
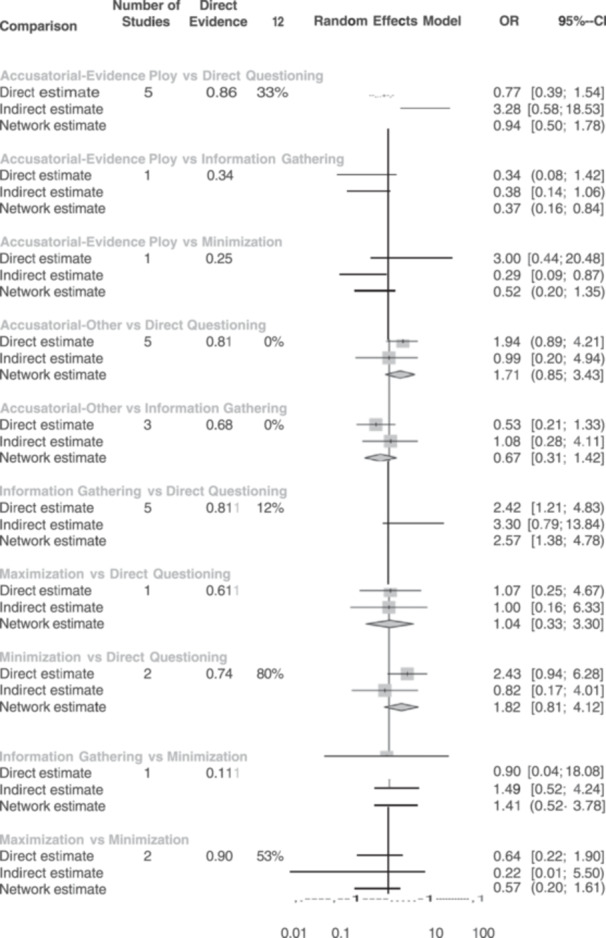
Forest plot for six‐node model: True confessions.

Figure 8 shows the network graph for the six‐node model of true confessions. Notice that not all nodes are connected by edges (lines). These are comparisons for which we have no direct evidence; no study directly compared these two conditions. Figure 9 shows the forest plot with direct, indirect, and combined effects. Note that the forest plot does not show indirect estimates for comparisons with no direct estimates. These are for different forms of the accusatorial approach, and these estimates are not our primary focus.

Ideally, each comparison would be estimated by at least two studies. That is not the case for this network. Four of the 10 edges with direct evidence for true confessions have only one effect size (Accusatorial‐Evidence Ploy vs. Information‐Gathering, Accusatorial‐Evidence Ploy vs. Minimization, Information‐Gathering vs. Minimization, and Maximization vs. Direct Questioning; see Figure 8). Most of the direct, indirect, and combined network estimated mean odds ratios are not statistically significant. The only significant effects are for (1) information‐gathering versus direct questioning, with the former resulting in more true confessions (combined OR = 2.57, 95% CI 1.38, 4.78) with reasonable consistency between the direct and indirect effects; and (2) accusatorial‐evidence ploy versus information‐gathering with the former resulting in fewer true confessions (combined OR = 0.37, 95% CI 0.16, 0.84). Three comparisons have direct and indirect estimates in opposite directions and of nontrivial size, albeit not statistically significant. These inconsistencies are for accusatorial‐evidence ploy versus direct questioning (direct OR = 0.77, indirect OR = 3.28), accusatorial‐evidence ploy versus minimization (direct OR = 3.00, indirect OR = 0.29), and minimization versus direct questioning (direct OR = 2.43, indirect OR = 0.82). Note that the confidence intervals for each of these differences overlap. As such, these differences may simply reflect sampling error and, more generally, indicate a lack of precision in estimating these effects, given the small number of effect sizes estimating these comparisons.

For true confessions, neither *Q* statistic (see Table 5) is significant for this model. Overall, the network rankings suggest that information‐gathering has the largest effect, with a *p*‐score of 0.90, consistent with the three‐node model. However, both accusatorial approaches of minimization and other approaches have a greater than 50% chance of being top‐rated (*p*‐scores of 0.70 and 0.64, respectively). Direct questioning and the other two accusatorial approaches, maximization and evidence‐ploy, are likely top‐ranked between a quarter and a third (Table 6).

**Table 5:** Test of heterogeneity (within designs) and consistency (between designs).

Source	*Q*	*df*	*p*	*τ* ^2^	_*I*_2
3 Node/True confessions				0.2890	40
Total	28.09	17	0.044		
Within designs	26.90	14	0.020		
Between designs	1.19	3	0.756		
3 Node/False confessions				0.7825	66
Total	77.58	26	0.000		
Within designs	74.41	23	0.000		
Between designs	3.16	3	0.367		
6 Node/True confessions				0.2369	33
Total	25.17	17	0.091		
Within designs	9.78	8	0.281		
Between designs	15.40	9	0.081		
6 Node/False confessions				0.7951	68
Total	86.42	28	0.000		
Within designs	32.19	17	0.014		
Between designs	54.23	11	0.000		

**Table 6:** Network ranking table for six‐node models.

Outcome	Condition	*p*‐Score
True confessions	Information Gathering	0.9
	Minimization	0.70
	Accusatorial‐Other	0.64
	Maximization	0.31
	Accusatorial‐Evidence Ploy	0.24
	Direct Questioning	0.21
False confessions	Information Gathering	0.93
	Direct Questioning	0.78
	Maximization	0.59
	Accusatorial‐Other	0.28
	Minimization	0.22
	Accusatorial‐Evidence Ploy	0.21

*Note*: Higher odds ratio “good” for true confessions and lower odds ratio “good” for false confessions.

###### False confessions

**Figure 10:**
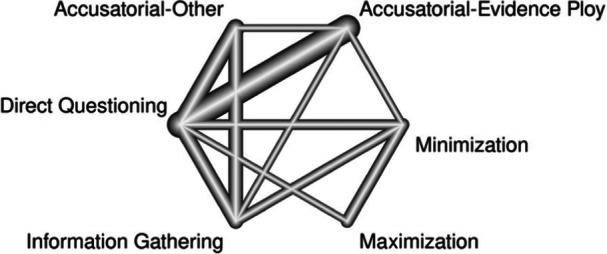
Network graph for six‐node model: False confessions.

Figure 10 shows the network graph for the six‐node model of false confessions. There are more odds ratios overall for false confession, with eleven comparisons with direct estimates. However, four also have only one estimate (Accusatorial‐Evidence Ploy vs. Accusatorial‐Other, Accusatorial‐Evidence Ploy vs. Information‐Gathering, Accusatorial‐Evidence Ploy vs. Minimization, Maximization vs. Direct Questioning).

By and large, the results from the six‐node model suggest that accusatorial approaches result in more false confessions. We found significant effects for (1) accusatorial‐evidence ploy versus direct questioning, with the former resulting in more false confessions (combined OR = 2.98, 95% CI 1.59, 5.59); (2) accusatorial‐evidence ploy versus information‐gathering with the former resulting in more false confessions (combined OR = 4.47, 95% CI 1.46, 13.68), though this is largely driven by the indirect effect and has a very large confidence interval limiting our confidence in this particular finding; (3) accusatorial‐other versus direct questioning with the former resulting in more false confessions (combined OR = 3.12, 95% CI 1.37, 7.10); (4) accusatorial‐other versus information‐gathering with the former resulting in more false confessions (combined OR = 4.67, 95% CI 1.61, 13.55); and (5) information‐gathering versus minimization with the latter resulting in more false confessions (combined OR = 0.25, 95% CI = 0.08, 0.83), largely driven by the indirect effect. Two comparisons have direct and indirect estimates in opposite directions, albeit not statistically significant. These inconsistencies are for accusatorial‐evidence ploy versus accusatorial‐other (direct OR = 1.23, indirect OR = 0.88) and accusatorial‐evidence ploy versus minimization (direct OR = 0.35, indirect OR = 1.91).

**Figure 11:**
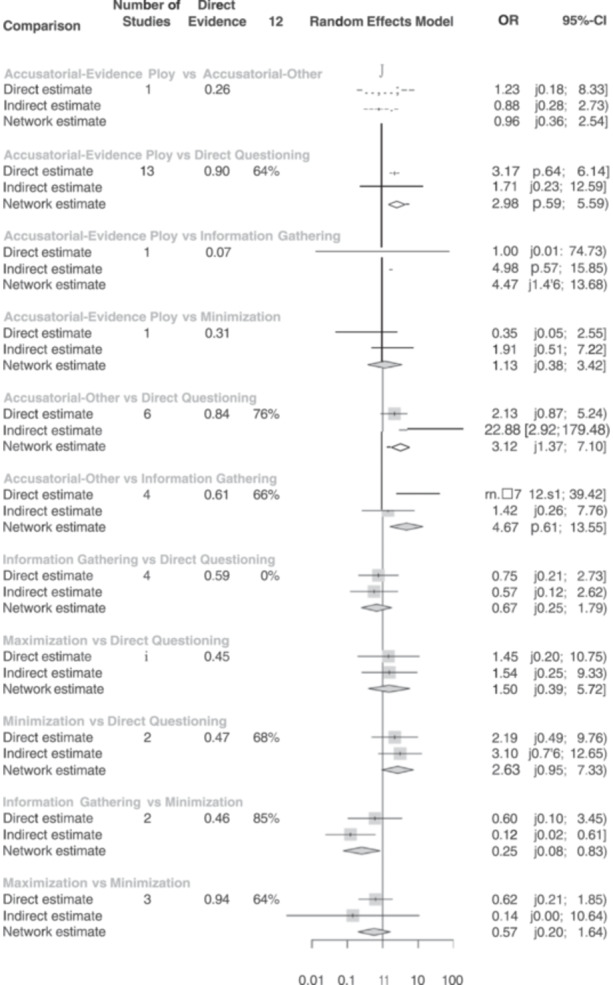
Forest plot for six‐node model: False confessions.

Not surprisingly, given the visual pattern of evidence in the forest plots for the false confession model (Figure 11), the *Q* statistic between designs is statistically significant, supporting the conclusion that the network is inconsistent (see Table 5). The within designs *Q* is also statistically significant, indicating heterogeneity in the odds ratios within comparisons. Thus, we need to interpret the relative rankings shown in Table 6 cautiously.

The relative rankings for false confessions suggest that information‐gathering has a 93% probability of being best at reducing the number of false confessions, followed by direct questioning with a *p*‐score of 0.78. However, the accusatorial maximization approach has a slightly greater than 50% chance of also being best at reducing false confessions, with a *p*‐score of 0.59. This reflects the relatively large confidence intervals around these estimates. The other three accusatorial approaches are less likely to be the best for reducing false confessions, each with a *p*‐score of 0.28 or less.

#### 6.3.2 Moderator analysis

It is not possible to conduct a moderator analysis within the frequentist statistical framework implemented in the *netmeta* package. It is possible, however, to do so within a Bayesian framework. We opted to conduct the moderator analyses at the level of contrast pairs rather than across the network as a whole. We do so because our network is sparse, and our moderator variables do not vary within each node. We also restricted our moderator analyses to contrast pairs with ten or more odds ratios on the two three‐node models. This restricted our moderator analyses to the contrast between accusatorial and direct questioning experimental conditions for true confessions (12 pairs or odds ratio) and false confessions (20 pairs or odds ratios).

Table 7 shows the results of the moderator analyses. Each row is a separate model. The first moderator analysis of interest is whether the cheating or alt‐key paradigms was used in the study. The two studies that used something other than one of these two paradigms were dropped from this analysis. The model shows that the cheating paradigm produces a slightly lower mean logged odds ratio for false confessions (*b* = −0.25) compared to the alt‐key paradigm, although this difference is neither substantively meaningful in size nor statistically significant.

**Table 7:** Moderator analyses for accusatorial versus direct questioning, three‐node models.

			95% CI			
Model	*k*	*b*	Lower	Upper	*p*	*τ* ^2^
*False confessions*						
Paradigm (cheating vs. ALT‐key)	18	−0.25	−1.25	0.75	0.619	0.598
Randomly assign to guilt status	12	0.59	−0.66	1.85	0.354	0
Interrogators blind to guilt status	20	−0.15	−1.09	0.79	0.754	0.571
Violations to random assignment	20	−0.12	−1.05	0.81	0.803	0.575
Reported subject suspicious	20	0.02	−0.95	0.98	0.975	0.583
Differences between mock interrogators	20	0.14	−0.93	1.22	0.793	0.589
*True confessions*						
Randomly assign to guilt status	12	−0.25	−1.38	0.88	0.664	0.446
Interrogators blind to guilt status	12	−0.5	−1.52	0.52	0.339	0.375
Violations to random assignment	12	−0.44	−1.47	0.58	0.394	0.389
Reported subject suspicious	12	−0.73	−1.7	0.24	0.142	0.32
Differences between mock interrogators	12	−0.33	−1.36	0.7	0.532	0.418

*Note*: Each row is a separate meta‐regression model estimated via REML. The regression coefficient is a dummy code with “Yes” = 1.

The remaining moderator analyses all examine risk‐of‐bias items. None of these moderator variables explained significant variation in logged odds ratios across studies and, as such, are not meaningful explanations for the observed heterogeneity. However, it is worth noting that the direction of effect for true confessions is smaller (less desirable) odds ratios for the more rigorous studies. All of these regression coefficients are of a meaningful magnitude, albeit with large confidence intervals that extend into the positive region. For false confessions, the coefficients are smaller and mixed in terms of the direction of effect. Overall, not much can be inferred from these results.

#### 6.3.3 Publication bias

**Figure 12:**
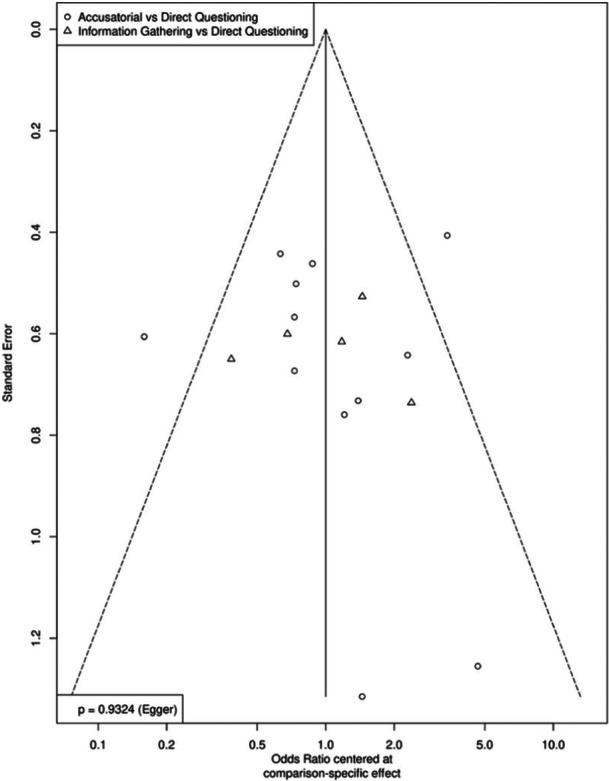
Funnel plot for three‐node model: True confessions.

**Figure 13:**
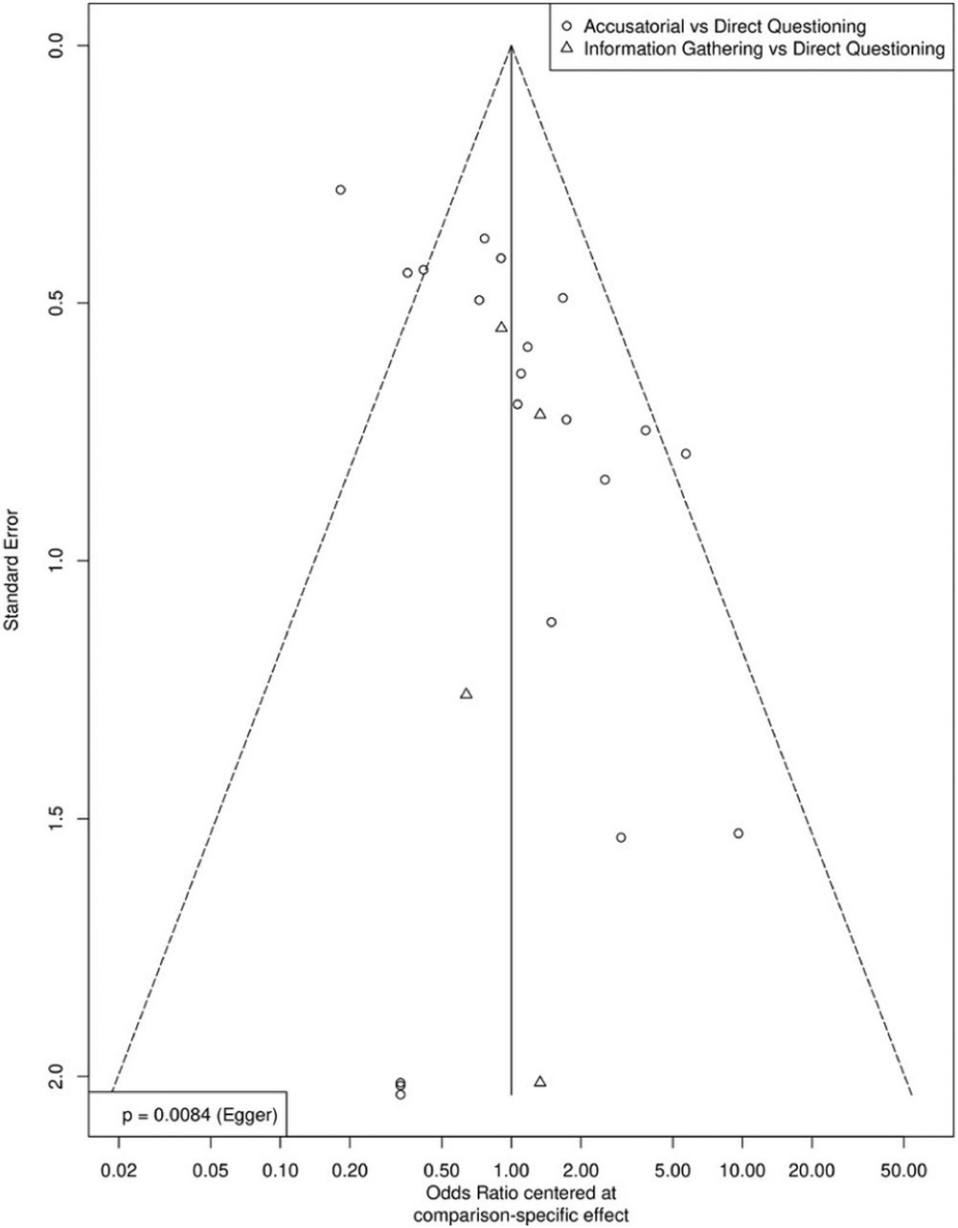
Funnel plot for three‐node model: False confessions.

We assessed for publication selection bias through a visual inspection of funnel plots for the two three‐node networks and through Egger's test for funnel plot asymmetry. The funnel plots are shown in Figures 12 and 13 for true and false confessions, respectively. Note that these funnel plots center the logged odds ratio around the mean for its contrast pair, enabling an assessment of publication bias for the entire network. The funnel plot for true confessions appears roughly symmetrical, consistent with the nonsignificant Egger's test (*p* = 0.9324). In contrast, the funnel plot for false confessions is asymmetrical and has a significant Egger's test (*p* = 0.008). This suggests the possibility of publication selection bias for results from the false confessions network. Visually, this appears to be driven by the contrast between the accusatory approach and direct questioning. Thus, the mean odds ratio for this contrast may be upwardly biased.

A complete summary of results can be found in Summary of findings Table 1.

## 7 DISCUSSION

### 7.1 Summary of main results

The primary purpose of the current research was to update and extend a meta‐analysis conducted by Meissner et al. (2012; see also, Meissner et al., 2014) which compared accusatorial and information‐gathering approaches in interviewing and interrogation. In the original report, Meissner and colleagues (2012) found that, compared to both accusatorial and control (direct questioning/no tactic) approaches, information‐gathering approaches resulted in significantly more true confessions. Accusatorial approaches, however, also produced significantly more true confessions compared to control approaches. That being said, accusatorial approaches also produced significantly more false confessions when compared to both direct questioning (control) and information‐gathering approaches, while information‐gathering approaches did not produce a similarly higher rate of false confessions compared to control approaches. Approximately 10 years after Meissner and colleagues' analysis, we were able to examine 29 experimental laboratory studies (more than doubling the number of studies they had examined) and conduct network meta‐analyses that allowed us to expand the categorization of interrogation approaches and take advantage of both direct and indirect relationships examined in the literature.

In updating the original analysis, we again found that an information‐gathering approach resulted in more true confessions than both an accusatorial (combined OR = 0.55, 95% CI 0.29, 1.05) and direct questioning approach (combined OR = 2.43, 95% CI 1.29, 4.59), though only the comparison with direct questioning was statistically significant. We also again found that an accusatorial approach resulted in significantly more false confessions compared to an information gathering (combined OR = 4.41, 95% CI 1.77, 10.97) and direct questioning approach (combined OR = 3.03, 95% CI 1.83, 5.02). The similarity in the direct, indirect, and network effects for these comparisons increases our certainty (see Summary of findings Table 1).

In expanding the original analysis, we did not find many differences in the rate of true confessions when examining specific accusatorial interrogation tactics. However, a network effect indicated that evidence ploys significantly reduced the number of true confessions compared to an information‐gathering approach. Of note, neither the direct nor indirect effects were significant for this contrast but the overall network effect was, demonstrating the additional information a network meta‐analysis can provide. Furthermore, the information‐gathering approach resulted in more true confessions in both the direct and network effects when compared to direct questioning. Net‐rankings suggest that information gathering is likely the best approach to produce true confessions, followed by minimization and other accusatorial tactics. When examining the six‐node network analysis for false confessions, there was significant heterogeneity both within and between designs, as evidenced by the highly dissimilar direct, indirect, and network estimates. Not surprisingly, then, the net‐ranking suggests that both information‐gathering and direct questioning approaches have a reasonable chance of being the best option for reducing false confessions.

### 7.2 Overall completeness and applicability of evidence

Overall, the current study highlights the need for high‐quality research investigating the influence of interviewing and interrogation methods and tactics on true and false confessions. Despite a substantial increase in the number of studies represented in our analyses, there remain clear areas of inquiry that are under‐studied (e.g., direct comparisons of information‐gathering and accusatorial approaches) that limit the field's ability to reach a consensus that would prove most helpful for policy and practice recommendations. In fact, our network analyses for both true and false confessions suggest that several contrasts did not include a direct estimate or included only a single effect size estimate. Therefore, we were limited in the analyses we could conduct concerning specific moderator analyses and risk of bias assessments. The lack of data comparing specific interrogation approaches has direct implications for the applicability of the evidence. Specifically, it may be more useful to initiate policy discussions that target specific tactics (e.g., minimization) as opposed to an entire school of interrogation (e.g., accusatorial approaches). That said, the available evidence is ideal for research and policy implications as the strength of experimental designs can speak directly to the impact of interrogation approaches on true and false confessions. Further, the majority of studies involved the use of deception with mock suspects in ways that increased the psychological realism and external validity of these experiments, and in turn, allowed for findings that speak to interrogation and interview settings.

In support of our claims regarding the need for additional research, publication bias assessments suggest a strong possibility that our review is missing evidence. There are several possible explanations for the likely publication bias: (1) our search did not uncover all available evidence; (2) the experimental manipulations in the literature lack strength; or (3) the publication bias assessment overestimates missingness. To the first point, we are confident in the search procedures we conducted and believe through our digital searches, manual searches, outreach efforts, and expertise in the field that we were able to capture the majority of relevant studies available in the extant literature. It is possible, however, that despite our efforts, researchers did not share pilot studies or abandoned laboratory studies that were deemed not relevant by the researchers we contacted. If such determinations were made, there is a possibility that the reason such studies were abandoned is because they were producing unexpected results. Based on the information available to us, it is not possible to ascertain the likelihood of this type of “file drawer” problem, speaking to the necessity of pre‐registering experimental studies within the framework of open science. The second possibility is that the design of the studies we included did not allow enough of an opportunity for null findings. Specifically, it could be the case that weak interrogation manipulations, lack of blinding, or some other form of systematic bias could have artificially influenced effect sizes. We also find these possibilities less compelling as our risk of bias items suggests that researchers employed as much psychological realism as possible and double‐blinded where appropriate. Furthermore, physiological studies suggest that participants experience meaningful levels of stress during mock interrogations and that stress levels systematically vary based on the interrogation approach, even in an experimental setting (Guyll et al., 2019; Normile & Scherr, 2018). Thus, insofar as physiological reactivity is evidence of participants' investment in the study, it is possible to use laboratory studies as an analog to real‐world interrogations. Finally, publication bias may be in effect, but not to the extent estimated by the available analyses.

### 7.3 Quality of the evidence

As part of our eligibility requirements, we only included studies that experimentally manipulated the interrogation approach. A common criticism about experimental studies is that they lack external validity as people question whether a threat of academic sanctions, for example, can adequately compare to the stress of a criminal accusation. Depending on the country and suspect, an interrogation in the real world is likely to occur under very different circumstances to the laboratory. For example, the room in which an individual is questioned, whether an attorney or supportive person is present during questioning, and the rights an individual has to leave the situation or not speak when questioned differs between a relatively low‐stakes experiment and an actual interrogation. All of these differences could impact external validity as participants in an experiment might not behave like suspects in an actual interrogation. That said, there is reason to believe laboratory studies can be used as an analog to real‐world interrogations. First, research has demonstrated that students in laboratory settings also experience a sensation of custodial custody in that they do not feel free to leave the lab‐based interrogation (Alceste et al., 2018)—an effect that has been replicated using virtual reality (Guarnera & Cleary, 2024). Second, in addition to the physiological stress reactions previously mentioned (Guyll et al., 2019; Normile & Scherr, 2018), a recent effort has found that when the cheating paradigm is modified to include the threat of legal consequences, similar confession trends are found compared to the original cheating paradigm that used a threat of academic consequences (Houston & Russano, 2024). Finally, most of the studies bolstered psychological realism by using deception to convince unsuspecting participants of the seriousness of the accusation and subsequent consequences. Together, these factors reduce threats to internal validity and increase external validity. Beyond the experimental designs and efforts towards psychological realism, almost all the studies used random assignment for participants' guilt status, blinded experimenters to participants' guilt status where possible, and did not engage in selective reporting of confession outcomes. Thus, the quality of the evidence available is starting at a high level.

A few items, however, raise potential concerns about the quality of evidence available for the current review. First, authors did not consistently report if they investigated adherence to randomization, whether participants were suspicious, or differences in confession rates between experimenters. It is difficult to comment on this point because no authors explicitly stated that they did not explore these potential complications. It is possible that all included studies involved rigorous exploration and treatment of their data but failed to include these details in their reporting of results. Other evidence, however, suggests that the field may not be consistent, whether reported or not, in their handling of cases that violate randomization to guilt conditions (Redlich et al., 2023). If the same is true regarding suspicious participants or experimenter differences, there would be direct implications for the quality of the evidence—though possibly of minimal impact on outcomes (see Redlich et al., 2023). Unfortunately, current reporting practices prohibited us from evaluating these possibilities. We recommend increased transparency in future reporting and utilization of open science practices.

In addition, our reading of the literature suggests some inconsistency in the terminology used to describe the interrogation approaches used in experimental work. Specifically, the majority of researchers referred to their approach broadly (e.g., minimization) without including explicit statements about whether interrogations were scripted or what precisely was said to capture a specific approach (see Kelly et al., 2019). This presented several problems: (1) by broadly referring to an approach, it was difficult to determine if participants were exposed to one or multiple tactics under the macro‐umbrella term; (2) it was not possible to compare between studies to determine if researchers were conceptualizing a tactic or approach in the same way across studies; and (3) in our analysis and conclusions, we were forced to rely on broader terms (e.g., maximization) instead of a more nuanced examination (e.g., guilt induction vs. exaggerating consequences). Although research has identified over 70 individual tactics (Kelly et al., 2013), the limited experimental research only allowed us to focus on these broad, macro‐level interrogation tactics. While our network analysis allowed for a more nuanced examination of accusatorial tactics than was possible a decade ago, like Meissner et al. (2012), we encourage additional research to fully understand how interrogation tactics impact confession rates.

### 7.4 Potential biases in the review process

It is worth noting that all the reviewers involved in the current effort are U.S.‐based and there is a possibility that, as such, we were less likely to capture research conducted outside of the United States. To minimize a potential U.S. bias, we did not limit our electronic search by location, we specifically included non‐U.S. researchers in our outreach efforts, and we searched databases based in other countries. Though most of the research we found was conducted in the United States, we also found a meaningful number of studies conducted outside of the United States (e.g., Canada, Japan). To minimize potential bias in the screening or coding of studies, everything was coded by two independent coders, and disagreements were resolved via discussion. It is also worth noting that both coders were very familiar with the interrogation literature. As such, there is a possibility that they made assumptions about information presented in manuscripts that a naive reader would not have. We attempted to mitigate this risk by including an expert in meta‐analysis, who is not a content expert, in the formation of our coding protocols and the analysis of data.

### 7.5 Agreements and disagreements with other studies or reviews

As shown in Table 8, our results generally agree with the original findings reported by Meissner et al. (2014). More specifically, there are four noteworthy points of convergence. First, both reports found that information‐gathering approaches significantly increase true confessions and seem to decrease false confessions (albeit n.s. in the current analysis) when compared with direct questioning. Second, both reports determined that accusatorial approaches significantly increase the rates of false confessions when compared to information‐gathering approaches; however, unlike the original analysis, our review did not find a significant impact on true confessions. Third, both reports found that accusatorial approaches also significantly increase the risk of false confessions when compared to direct questioning. Again, however, the original analyses suggested that accusatorial tactics also increase true confessions, an effect that did not reach significance in the updated review. Together, these convergent findings lend support for moving away from accusatorial methods to minimize false confessions. If countries like the U.S. are not yet ready or able to take the same step away from accusatorial approaches that places like the U.K. have already taken, policymakers may have more success targeting particularly problematic tactics like false evidence ploys and minimization. Indeed, seven states have banned lying to juveniles during interrogation, with several other states now considering similar legislation (Kassin et al., 2024). It is also worth noting that the magnitude of effects is generally smaller in the updated review, though always in the same direction, when directly compared to the original review. Finally, comparing confidence intervals between the original and updated meta‐analyses reveals that all six confidence intervals overlap. In fact, with the exception of the false confession contrast between accusatorial and direct questioning approaches, each direct estimate in the current analysis falls within the confidence intervals found in the original analyses.

**Table 8:** Comparison between current results and previous meta‐analysis.

Interrogative contrast	Study	Outcome	Hedge's *g*	95% CI	
				LL	UL
Information‐Gathering versus Direct Questioning	Current	True confession	0.23*	0.04	0.42
		False confession	−0.08	−0.41	0.26
	Meissner et al.	True confession	0.67*	0.02	1.32
		False confession	−0.23	−0.98	0.52
Accusatorial versus Information‐Gathering	Current	True confession	−0.18	−0.39	0.02
		False confession	0.41*	0.13	0.68
	Meissner et al.	True confession	−0.64*	−1.28	−0.01
		False confession	0.77*	0.08	1.46
Accusatorial versus Direct Questioning	Current	True confession	0.08	−0.04	0.21
		False confession	0.29*	0.16	0.43
	Meissner et al.	True confession	0.46*	0.06	0.86
		False confession	0.74*	0.35	1.12

*Note*: The current meta‐analyses were conducted using logged odds as the effect size estimate. We converted direct estimates from the three‐node network analysis to Hedge's g using the Cox logit for an easier comparison with the original review. All effect sizes are presented such that positive numbers indicate that the first approach in the contrast (e.g., Information‐Gathering in the Information‐Gathering vs. Direct Questioning) is more likely to produce either true or false confessions.

In line with Meissner et al. (2012) and another methodological review of interrogation literature (Stewart et al., 2018), we also assessed the impact of research paradigm on confession rates. Our analysis suggested that the paradigm did not meaningfully nor significantly impact confession rates, which aligns with the conclusions reached by Meissner et al. (2012). However, Stewart et al. (2018) found that alt‐key studies elicited more false confessions than cheating paradigm studies across 24 studies. Though a direct comparison cannot be made between our findings and Stewart et al. (2018) because of the differences in eligibility criteria, it is an issue worthy of further consideration by researchers.

## 8 AUTHORS' CONCLUSIONS

### 8.1 Implications for practice and policy

False confessions are undesirable outcomes in many ways: the false confessor is more likely to be wrongly convicted and incarcerated (see Scherr et al., 2020); the integrity of our criminal legal system is compromised; and the chances of allowing the true perpetrator to remain free to commit additional crimes are heightened (Norris et al., 2020). The current analysis suggests that information‐gathering approaches can be more effective in eliciting confessions when compared with accusatorial methods, but also have the advantage of eliciting more diagnostic information. Specifically, in the studies examined here, information‐gathering approaches produced more true confessions, whereas accusatorial approaches produced significantly more false confessions. As such, the current analysis suggests that law enforcement, military, and intelligence agencies that rely on accusatorial approaches should adopt the use of information‐gathering approaches to interrogation to increase the likelihood of obtaining diagnostic outcomes.

Providing more support for a move away from accusatorial approaches, our analyses suggest that evidence ploys may produce fewer true confessions than information‐gathering approaches, but more false confessions compared to both information‐gathering and direct questioning approaches. There has been some movement on this issue since 2021, as several states (e.g., Illinois, Oregon, Utah) have banned the use of deception, such as the use of false evidence ploys, in interrogations with minors. The ban of deception with minors is a promising first step and potentially has found success as it targets a specific tactic. However, our studies were not limited to minors and suggest that accusatorial approaches generally do not perform as well as alternative approaches; thus, we offer evidence that it would be advantageous to avoid accusatorial approaches with suspects of any age. Countries like the United States that rely on accusatorial approaches may also benefit from consulting efforts like the Police and Criminal Evidence Act (PACE) from the United Kingdom, which, in addition to relying on information‐gathering approaches, provides several safeguards for preventing false confessions and subsequent wrongful convictions by, for example, requiring the recording of interrogations.

### 8.2 Implications for research

In accomplishing this systematic review, it became clear to us that the current experimental literature must continue to mature if we are to offer a complete understanding of the various psychological, sociological, criminological, and cultural factors that influence the interrogative process. First, to offer more directed policy recommendations concerning accusatorial methods, additional research comparing accusatorial tactics is needed. Second, while we have a more robust understanding of the factors that lead to false confessions in an accusatorial interrogative context (see Kassin et al., 2010), existing literature assessing the value of alternative methods of interrogation that might promote the diagnostic elicitation of confession evidence in the law enforcement context (see Meissner et al., 2015, 2010), or the elicitation of critical knowledge in a military or intelligence context (see Evans et al., 2010; Redlich, 2007) is limited.

Second, compared to a decade ago, we have more evidence concerning information‐gathering approaches; however, there is little evidence about different types of information‐gathering approaches (e.g., cognitive interviews) or how rapport‐ versus trust‐based approaches may influence confessions (see Oleszkiewicz et al., 2024). Furthermore, we would suggest that researchers consider both binary confession and information yield as outcomes for experimental work, as both outcomes provide meaningful evidence about the validity of interviewing and interrogation approaches. By considering both potential outcomes of suspect questioning, our ability to compare information yield and confessions will improve and allow for more direct comparisons between these two lines of research.

Finally, beyond a general call for more research comparing interrogation and interview techniques on confessions, the results of our analysis suggest a greater need for transparency in reporting. Our risk of bias items suggest that manuscripts often do not provide sufficient information to fully appreciate the interrogation tactic under investigation or the strength of the research design. We suggest that future researchers pre‐register their studies and make both study materials and analyses openly available for readers to evaluate in line with recent calls for open science.

Whereas research over the past three decades or so has provided invaluable information about the circumstances under which suspects admit guilt, overall, we echo Meissner and colleagues' (2014) sentiment from almost a decade ago that there remains much to learn about when and why people truly and falsely confess in interrogations.

## CONTRIBUTIONS OF AUTHORS

Mary Catlin is an advanced graduate student being supervised by Professor Redlich. She has assisted Professor Redlich with another meta‐analysis (Redlich et al., 2023) and has previously published on perceptions of false admissions of guilt in addition to substantial experience managing studies using the cheating paradigm (Russano, Meissner, et al., 2005). Mary has also conducted a pilot test of the updated meta‐analysis outlined above as part of a class assignment for a meta‐analysis course taught by Professor Wilson, who is an expert in meta‐analysis. Professor Wilson has written one of the most widely used texts on meta‐analysis (Lipsey & Wilson, 2001), serves as the methods editor for the Crime and Justice Group for the Campbell Collaboration, is an associate editor for Research Synthesis Methods, and was awarded the Frederick Mosteller Award for Distinctive Contributions to Systematic Reviewing. Furthermore, Professor Wilson has produced several systematic reviews within the field of criminology (e.g., Wilson et al., 2018, 2019) and has written on the utility of network meta‐analyses with recommendations for Campbell reviews specifically (Wilson et al., 2016).

Both Professors Redlich and Meissner are content experts and conducted the original review. Professor Redlich has conducted several interviewing and interrogation studies, and has reviewed the literature on U.S. police and military interrogations, notably as part of the editing team for two volumes outlining investigative interviewing and interrogation internationally (Walsh et al., 2016a, 2016b). Furthermore, Professor Redlich was involved in the American Psychology – Law Society's scientific review committee's scientific review paper on police interrogations and false confessions (see Kassin et al., 2010). Professor Meissner has evaluated the deception detection and interviewing/interrogation literatures, including conducting several meta‐analyses in this area (Meissner & Kassin, 2002; Meissner et al., 2017; Snook et al., 2021). He has also co‐organized a conference sponsored by the American Psychological Association on investigative interviewing. This conference developed into a co‐edited volume entitled, *Interrogations and confessions: Current research, practice, and policy recommendations*, which was published by the American Psychological Association (Lassiter & Meissner, 2010). Furthermore, Professor Meissner was awarded the Excellence in Research Award from the International Investigative Interviewing Research Group, has been awarded a 3 year research grant from the U.S. Department of Justice to investigate the state of the interrogation literature and the dissemination of scientific knowledge in that area.

Talley Bettens is a graduate student supervised by Professor Redlich. She has assisted Professor Redlich with another meta‐analysis (Redlich et al., 2023) that required many of the same tasks/skills that were asked of her for the current endeavor. She has recently published work related to the interrogation of youth in schools, and the ramifications of confession evidence for both juveniles and adults.

Finally, both Sujeeta Bhatt and Susan Brandon contributed to the original review. Furthermore, Drs. Bhatt and Brandon served as program managers for research at the High‐Value Detainee Interrogation Group (Federal Bureau of Investigation). Under their leadership, a decade of research was conducted on best practices in interviewing and interrogation. In this context, Drs. Bhatt and Brandon have contributed significantly to the development of the research literature on science‐based approaches to investigative interviewing (Brandon & Meissner, 2023; Brandon et al., 2019).

Who is responsible for the below areas? Please list their names:

- Content: Mary Catlin and Allison Redlich
- Systematic review methods: Mary Catlin and David B. Wilson Statistical analysis: Mary Catlin and David B. Wilson
- Information retrieval: Mary Catlin and Christian Meissner Screening and coding: Mary Catlin and Talley Bettens
- Drafting of reports: Mary Catlin, Allison Redlich, David B. Wilson, and Christian Meissner Editing of reports: all authors

## DECLARATIONS OF INTEREST

None of the reviewers have financial conflicts of interest. Professor Meissner, Professor Redlich, and Drs. Brandon and Bhatt were authors on the original review which this update is based on. Furthermore, Professor Wilson was the Campbell editor who oversaw the original review.

### PLANS FOR UPDATING THIS REVIEW

The review will be updated every 5–10 years. These efforts will primarily be led by reviewers Meissner and Redlich, or their students.

## SOURCES OF SUPPORT

### Internal sources

- No sources of support provided.

### External sources

- High‐Value Detainee Interrogation Group, USA.

Funding for this project was provided by the Federal Bureau of Investigation's High‐Value Detainee Interrogation Group (research contract #15F06720C0001969). Any opinions, findings, conclusions, or recommendations expressed in this article are those of the authors and do not reflect the views of the U.S. government.

**What's new**

**Table:** 

Date	Event	Description
29 August 2023	New citation required but conclusions have not changed	Protocol published
2 March 2023	New search has been performed	Protocol published

## Supporting information

Supplementary material 1: CA000277‐SUP‐01‐searchStrategy.html Search strategies.

Supplementary material 2: CA000277‐SUP‐02‐characteristicsOfIncludedStudies.html Characteristics of included studies.

Supplementary material 3: CA000277‐SUP‐03‐characteristicsOfExcludedStudies.html Characteristics of excluded studies.

Supplementary material 5: CA000277‐SUP‐05‐other.html Individuals Contacted.

Supplementary material 6: CA000277‐SUP‐06‐other.html Coding Protocol.

Supplementary material 4: CA000277‐SUP‐04‐dataPackage.zip Data package.

## Footnotes

^1^
Note that we used the US spelling of maximization (maximiz*) in our search. This spelling could have limited our search. However, given the expanse of search terms used, the fact that most research on interrogations is conducted in the US, and maximization is a tactic used in US interrogation approaches, we believe any impact likely negligible.
