# Supplementary material for: Interview and interrogation methods and their effects on true and false confessions: A systematic review update and extension
Source: Campbell Syst Rev. 2024 Oct 10;20(4):e1441. doi: 10.1002/cl2.1441 (PMC11465838; doi:10.1002/cl2.1441)
Supplement: Supplementary file 1 — Supplementary material 1: CA000277‐SUP‐01‐searchStrategy.html Search strategies. [file CL2-20-e1441-s001.html]

Search strategies


# Supplementary material 1 to: Interview and Interrogation Methods and their Effects on True and False Confessions: An Update and Extension

Catlin M, Wilson D, Redlich AD, Bettens T, Meissner C, Bhatt S, Brandon S
  
https://doi.org/10.1002/14651858.CA000277

The material in this section has been supplied by the author(s) for publication under a Licence for Publication and the author(s) are solely responsible for the material. Cochrane has peer reviewed this material in accordance with its editorial policies, but Cochrane has not copyedited, formatted or proofread. Cochrane accordingly gives no representations or warranties of any kind in relation to, and accepts no liability for any reliance on or use of, such material.

Back to top

# Search strategies

## Search Strategies

*Note.* All searches were filted for results available in English.

|  |  |  |  |  |  |
| --- | --- | --- | --- | --- | --- |
| **Publisher Platform** | **Date of Search** | # | Searches | **Databases** | Results |
| ProQuest | October 20, 2022 | 1 | AB,TI,IF,CC(interrogat\* OR information OR inquisitorial OR interview\* OR accusat\* OR 'deception detection' OR PEACE OR PACE OR adversar\* OR REID OR minimi?\* OR maximiz\* OR 'cognitive interview\*' OR 'conversation management' OR 'ethical interview\*' OR 'strategic evidence' OR miranda OR coerc\* OR entrap\* OR responsib\* OR commit\*) | Australia & New Zealand Database, Criminal Justice Database, ERIC, ProQuest Dissertations & Theses Global, Psychology Database, Social Science Database, Sociological Abstracts, Sociology Database, UK & Ireland Database | 2635350 |
|  |  | 2 | AB,TI,IF,CC(random\* OR control OR comparison OR experiment\* OR RCT OR manipulat\* OR lab\* OR factorial OR effect) | Australia & New Zealand Database, Criminal Justice Database, ERIC, ProQuest Dissertations & Theses Global, Psychology Database, Social Science Database, Sociological Abstracts, Sociology Database, UK & Ireland Database | 4348167 |
|  |  | 3 | AB,TI,IF,CC(confess\* OR disclos\*) | Australia & New Zealand Database, Criminal Justice Database, ERIC, ProQuest Dissertations & Theses Global, Psychology Database, Social Science Database, Sociological Abstracts, Sociology Database, UK & Ireland Database | 77042 |
|  |  | 4 | AB,TI,IF,CC(suspect\* OR guilt\* OR innocen\*) | Australia & New Zealand Database, Criminal Justice Database, ERIC, ProQuest Dissertations & Theses Global, Psychology Database, Social Science Database, Sociological Abstracts, Sociology Database, UK & Ireland Database | 126325 |
|  |  | 5 | 1 and 2 and 3 and 4 | Australia & New Zealand Database, Criminal Justice Database, ERIC, ProQuest Dissertations & Theses Global, Psychology Database, Social Science Database, Sociological Abstracts, Sociology Database, UK & Ireland Database | 532 |
| EBSCOhost | October 20, 2022 | 1 | ( AB (interrogat\* OR information OR  inquisitorial OR interview\* OR accusat\* OR 'deception detection' OR PEACE OR PACE OR adversar\* OR REID OR minimi?\* OR maximiz\* OR 'cognitive interview\*' OR 'conversation management' OR 'ethical interview\*' OR 'strategic evidence' OR miranda OR coerc\* OR entrap\* OR responsib\* OR commit\*) ) OR ( TI (interrogat\* OR information OR inquisitorial OR interview\* OR accusat\* OR 'deception detection' OR PEACE OR PACE OR adversar\* OR REID OR minimi?\* OR maximiz\* OR 'cognitive interview\*' OR 'conversation management' OR 'ethical interview\*' OR 'strategic evidence' OR miranda OR coerc\* OR entrap\* OR responsib\* OR commit\*) ) OR ( SU (interrogat\* OR information OR inquisitorial OR interview\* OR accusat\* OR 'deception detection' OR PEACE OR PACE OR adversar\* OR REID OR minimi?\* OR maximiz\* OR 'cognitive interview\*' OR 'conversation management' OR 'ethical interview\*' OR 'strategic evidence' OR miranda OR coerc\* OR entrap\* OR responsib\* OR commit\*) ) | APA PsycExtra;APA PsycInfo;Criminal Justice Abstracts;National Criminal Justice Reference Service Abstracts;Psychology and Behavioral Sciences Collection | 1,689,237 |
|  |  | 2 | ( AB (random\* OR control OR comparison OR  experiment\* OR RCT OR manipulat\* OR lab\* OR factorial OR effect) ) OR ( TI (random\* OR control OR comparison OR experiment\* OR RCT OR manipulat\* OR lab\* OR factorial OR effect) ) OR ( SU (random\* OR control OR comparison OR experiment\* OR RCT OR manipulat\* OR lab\* OR factorial OR effect) ) | APA PsycExtra;APA PsycInfo;Criminal Justice Abstracts;National Criminal Justice Reference Service Abstracts;Psychology and Behavioral Sciences Collection | 3,017,649 |
|  |  | 3 | ( AB (confess\* OR disclos\*) ) OR ( TI  (confess\* OR disclos\*) ) OR ( SU (confess\* OR disclos\*) ) | APA PsycExtra;APA PsycInfo;Criminal Justice Abstracts;National Criminal Justice Reference Service Abstracts;Psychology and Behavioral Sciences Collection | 61,950 |
|  |  | 4 | ( AB (suspect\* OR guilt\* OR innocen\*) ) OR ( TI (suspect\* OR guilt\* OR  innocen\*) ) OR ( SU (suspect\* OR guilt\* OR innocen\*) ) | APA PsycExtra;APA PsycInfo;Criminal Justice Abstracts;National Criminal Justice Reference Service Abstracts;Psychology and Behavioral Sciences Collection | 85,131 |
|  |  | 5 | 1 and 2 and 3 and 4 | APA PsycExtra;APA PsycInfo;Criminal Justice Abstracts;National Criminal Justice Reference Service Abstracts;Psychology and Behavioral Sciences Collection | 873 |
| Web of Science | October 20, 2022 | 1 | interrogat\* OR information OR  inquisitorial OR interview\* OR accusat\* OR 'deception detection' OR PEACE OR PACE OR adversar\* OR REID OR minimi?\* OR maximiz\* OR 'cognitive interview\*' OR 'conversation management' OR 'ethical interview\*' OR 'strategic evidence' OR miranda OR coerc\* OR entrap\* OR responsib\* OR commit\* (Abstract) or interrogat\* OR information OR inquisitorial OR interview\* OR accusat\* OR 'deception detection' OR PEACE OR PACE OR adversar\* OR REID OR minimi?\* OR maximiz\* OR 'cognitive interview\*' OR 'conversation management' OR 'ethical interview\*' OR 'strategic evidence' OR miranda OR coerc\* OR entrap\* OR responsib\* OR commit\* (Title) or interrogat\* OR information OR inquisitorial OR interview\* OR accusat\* OR 'deception detection' OR PEACE OR PACE OR adversar\* OR REID OR minimi?\* OR maximiz\* OR 'cognitive interview\*' OR 'conversation management' OR 'ethical interview\*' OR 'strategic evidence' OR miranda OR coerc\* OR entrap\* OR responsib\* OR commit\* (Author Keywords) | Conference Proceedings Index: Social Sciences & Humanities, Social Science Citation Index | 1,436,272 |
|  |  | 2 | random\* OR control OR comparison OR  experiment\* OR RCT OR manipulat\* OR lab\* OR factorial OR effect (Abstract) or random\* OR control OR comparison OR  experiment\* OR RCT OR manipulat\* OR lab\* OR factorial OR effect (Title) or random\* OR control OR comparison OR  experiment\* OR RCT OR manipulat\* OR lab\* OR factorial OR effect (Author Keywords) | Conference Proceedings Index: Social Sciences & Humanities, Social Science Citation Index | 2,457,086 |
|  |  | 3 | confess\* OR disclos\* (Abstract) or confess\* OR disclos\* (Title) or confess\* OR disclos\* (Author Keywords) | Conference Proceedings Index: Social Sciences & Humanities, Social Science Citation Index | 46,615 |
|  |  | 4 | suspect\* OR guilt\* OR innocen\* (Abstract) or suspect\* OR guilt\* OR innocen\* (Title) or suspect\* OR guilt\* OR innocen\* (Author Keywords) | Conference Proceedings Index: Social Sciences & Humanities, Social Science Citation Index | 33,368 |
|  |  | 5 | 1 and 2 and 3 and 4 | Conference Proceedings Index: Social Sciences & Humanities, Social Science Citation Index | 282 |
| Other | October 27, 2022 | 1 | interrogation tactics and confessions | Google | 459,000 |
|  | October 27, 2022 | 1 | interrogation tactics and confessions | Google Scholar | 33,600 |
|  |  | 2 | Forward citation search | Google Scholar | na |
