# Supplementary material for: Interview and interrogation methods and their effects on true and false confessions: A systematic review update and extension
Source: Campbell Syst Rev. 2024 Oct 10;20(4):e1441. doi: 10.1002/cl2.1441 (PMC11465838; doi:10.1002/cl2.1441)
Supplement: Supplementary file 2 — Supplementary material 2: CA000277‐SUP‐02‐characteristicsOfIncludedStudies.html Characteristics of included studies. [file CL2-20-e1441-s005.html]

Characteristics of included studies


# Supplementary material 2 to: Interview and Interrogation Methods and their Effects on True and False Confessions: An Update and Extension

Catlin M, Wilson D, Redlich AD, Bettens T, Meissner C, Bhatt S, Brandon S
  
https://doi.org/10.1002/14651858.CA000277

The material in this section has been supplied by the author(s) for publication under a Licence for Publication and the author(s) are solely responsible for the material. Cochrane has reviewed this material, but Cochrane has not copyedited, formatted or proofread. Cochrane accordingly gives no representations or warranties of any kind in relation to, and accepts no liability for any reliance on or use of, such material.

Back to top

# Characteristics of included studies

## Table of contents

- Studies ordered by Study ID
  - Blair, 2007
  - Cole et al., 2013
  - Eastwood et al., 2020
  - Evans et al., 2013
  - Guyll et al., 2019
  - Hill et al., 2008
  - Huang and Teoh, 2019
  - Kassin and Kiechel, 1996
  - Klaver et al., 2008
  - Meissner et al., 2011
  - Narchet et al., 2011
  - Noc et al., 2023
  - Normile and Scherr, 2018
  - Normile et al., 2017
  - Paton et al., 2018
  - Perillo and Kassin, 2011
  - Redlich and Goodman, 2003
  - Rigoni, 2007
  - Russano et al., 2005a
  - Russano et al., 2005b
  - Smalarz et al., 2011
  - Swanner et al., 2010
  - Villalba, 2014
  - Wachi et al., 2018
  - Wilford and Wells, 2018
  - Woestehoff, 2016
  - Wright, 2013
- References to studies

## Studies ordered by Study ID

Blair, 2007

| ***Risk of bias*** | | |
| **Bias** | **Authors' judgement** | **Support for judgement** |

Cole et al., 2013

| ***Risk of bias*** | | |
| **Bias** | **Authors' judgement** | **Support for judgement** |

Eastwood et al., 2020

| ***Risk of bias*** | | |
| **Bias** | **Authors' judgement** | **Support for judgement** |

Evans et al., 2013

| ***Risk of bias*** | | |
| **Bias** | **Authors' judgement** | **Support for judgement** |

Guyll et al., 2019

| ***Risk of bias*** | | |
| **Bias** | **Authors' judgement** | **Support for judgement** |

Hill et al., 2008

| ***Risk of bias*** | | |
| **Bias** | **Authors' judgement** | **Support for judgement** |

Huang and Teoh, 2019

| ***Risk of bias*** | | |
| **Bias** | **Authors' judgement** | **Support for judgement** |

Kassin and Kiechel, 1996

| ***Risk of bias*** | | |
| **Bias** | **Authors' judgement** | **Support for judgement** |

Klaver et al., 2008

| ***Risk of bias*** | | |
| **Bias** | **Authors' judgement** | **Support for judgement** |

Meissner et al., 2011

| ***Risk of bias*** | | |
| **Bias** | **Authors' judgement** | **Support for judgement** |

Narchet et al., 2011

| ***Risk of bias*** | | |
| **Bias** | **Authors' judgement** | **Support for judgement** |

Noc et al., 2023

| ***Risk of bias*** | | |
| **Bias** | **Authors' judgement** | **Support for judgement** |

Normile and Scherr, 2018

| ***Risk of bias*** | | |
| **Bias** | **Authors' judgement** | **Support for judgement** |

Normile et al., 2017

| ***Risk of bias*** | | |
| **Bias** | **Authors' judgement** | **Support for judgement** |

Paton et al., 2018

| ***Risk of bias*** | | |
| **Bias** | **Authors' judgement** | **Support for judgement** |

Perillo and Kassin, 2011

| ***Risk of bias*** | | |
| **Bias** | **Authors' judgement** | **Support for judgement** |

Redlich and Goodman, 2003

| ***Risk of bias*** | | |
| **Bias** | **Authors' judgement** | **Support for judgement** |

Rigoni, 2007

| ***Risk of bias*** | | |
| **Bias** | **Authors' judgement** | **Support for judgement** |

Russano et al., 2005a

| ***Risk of bias*** | | |
| **Bias** | **Authors' judgement** | **Support for judgement** |

Russano et al., 2005b

| ***Risk of bias*** | | |
| **Bias** | **Authors' judgement** | **Support for judgement** |

Smalarz et al., 2011

| ***Risk of bias*** | | |
| **Bias** | **Authors' judgement** | **Support for judgement** |

Swanner et al., 2010

| ***Risk of bias*** | | |
| **Bias** | **Authors' judgement** | **Support for judgement** |

Villalba, 2014

| ***Risk of bias*** | | |
| **Bias** | **Authors' judgement** | **Support for judgement** |

Wachi et al., 2018

| ***Risk of bias*** | | |
| **Bias** | **Authors' judgement** | **Support for judgement** |

Wilford and Wells, 2018

| ***Risk of bias*** | | |
| **Bias** | **Authors' judgement** | **Support for judgement** |

Woestehoff, 2016

| ***Risk of bias*** | | |
| **Bias** | **Authors' judgement** | **Support for judgement** |

Wright, 2013

| ***Risk of bias*** | | |
| **Bias** | **Authors' judgement** | **Support for judgement** |

## References to studies

### Blair, 2007 {published data only}

- Blair, J P. The roles of interrogation, perception, and individual differences in producing compliant false confessions. Psychology, Crime & Law 2007;13(2):173-186. [DOI: 10.1080/10683160600632801]

### Cole et al., 2013 {published data only}

- Cole, T, Teboul, J C B, Zulawski, D E, Wicklander, D E, & Sturman, S G. False confessions and the use of incriminating evidence. Linguistice Evidence in Security, Law and Intelligence 2013;1(1):67-75. [DOI: 10.5195/lesli.2013.4]

### Eastwood et al., 2020 {published data only}

- Eastwood, J, Dunk, M, & Akca, D. Assessing the diagnosticity of a persuasion-based and a dialogue-based interrogation approach. Journal of Police and Criminal Psychology 2022;37(1):569-575. [DOI: 10.1007/s11896-020-09410-1]

### Evans et al., 2013 {published data only}

- Evans, J R, Meissner, C A, Ross, A B, Houston, K A, Russano, M B, & Horgan, A J. Obtaining guilty knowledge in human intelligence interrogations: Comparing accusatorial and information-gathering approaches with a novel experimental paradigm. Journal of applied research in memory and cognition 2013;2(2):83-88. [DOI: 10.1016/j.jarmac.2013.03.002]

### Guyll et al., 2019 {published data only}

- Guyll, M, Yang, Y, Madon, S, Smalarz, L, & Lannin, D G. Mobilization and resistance in response to interrogation threat. Law and Human Behavior 2019;43(3):307-318. [DOI: 10.1037/lhb0000337]

### Hill et al., 2008 {published data only}

- Hill, C, Memon, A, & McGeorge, P. The role of confirmation bias in suspect interviews: A systemic evaluation . Legal and Criminological Psychology 2008;13(2):357-371. [DOI: 10.1348/135532507X238682]

### Huang and Teoh, 2019 {published data only}

- Huang, K J, & Teoh, Y S. Rapport building in suspect interviewing: A comparison of relationship- and procedure-based approaches in laboratory setting. Psychology, Public Policy, and Law 2019;25(4):253-265. [DOI: 10.1037/law0000209]

### Kassin and Kiechel, 1996 {published data only}

- Kassin, S M, & Kiechel, K L. The social psychology of false confessions: Compliance, internalization, and confabulation. Psychological Science 1996;7(3):125-128. [DOI: 10.1111/j.1467-9280.1996.tb00344.x]

### Klaver et al., 2008 {published data only}

- Klaver, J R, Lee, Z, & Rose, V G. Effects of personality, interrogation techniques and plausibility in an experimental false confession paradigm. Legal and Criminological Psychology 2008;13(1):71-88. [DOI: 10.1348/135532507X193051]

### Meissner et al., 2011 {unpublished data only}

- Meissner, C A, Russano, M B, Rigoni, M E, & Horgan, A J . Is it time for a revolution in the interrogation room? Empirically validating inquisitorial methods. Unpublished data and materials from lead author 2011.

### Narchet et al., 2011 {published data only}

- Narchet, F M, Meissner, C A, & Russano, M B. Modeling the influence of investigator bias on the elicitation of true and false confessions. Law and Human Behavior 2011;35(6):452-465. [DOI: 10.1007/s10979-010-9257-x]

### Noc et al., 2023 {published data only}

- Noc, M, Ginet, M, & Deslauriers-Varin, N. False confession in innocent suspects: A look at the cognitive interview for suspects. Journal of Police and Criminology Psychology 2023;38:186-198. [DOI: 10.1007/s11896-022-09543-5]

### Normile and Scherr, 2018 {published data only}

- Normile, C J, & Scherr, K C. Police tactics and guilt status uniquely influence suspects' physiologic reactivity and resistance to confess. Law and Human Behavior 2018;42(6):497-506. [DOI: 10.1037/lhb0000306]

### Normile et al., 2017 {unpublished data only}

- Normile, C J, Scherr, K C, Madon, S, & Catlin, M. Does interrogation tactic order influence confession diagnosticity? Unpublished data from lead author 2017.

### Paton et al., 2018 {published data only}

- Paton, W, Bain, S A, Gozna, L, Gilchrist, E, Heim, D, Gardner, E, Cairns, D, McGranaghan, P, & Fischer, R. The combined effects of questioning technique and interviewer manner on false confessions. Journal of Investigative Psychology and Offender Profiling 2018;15(3):335-349. [DOI: 10.1002/jip.1513]

### Perillo and Kassin, 2011 {published data only}

- Perillo, J T, & Kassin, S M. Inside interrogation: The lie, the bluff, and false confessions. Law and Human Behavior 2011;35(4):327-337. [DOI: 10.1007/s10979-010-9244-2]

### Redlich and Goodman, 2003 {published data only}

- Redlich, A D, & Goodman, G S. Taking responsibility for an act not committed: The influence of age and suggestibility. Law and Human Behavior 2003;27(2):141-156. [DOI: 10.1023/A:1022543012851]

### Rigoni, 2007 {published data only}

- Rigoni, M E. Is it time for a revolutionary technique in the interrogation room? Empirically validating the influence of inquisitorial techniques on true and false confessions. Unpublished thesis (University of Texas at El Paso) 2007.

### Russano et al., 2005a {published data only}

- Russano, M B, Meissner, C A, Narchet, F M, & Kassin, S M. Investigating true and false confessions within a novel experimental paradigm. Psychological Science 2005;16(6):481-486. [DOI: 10.1111/j.0956-7976.2005.01560.x]

### Russano et al., 2005b {unpublished data only}

- Russano, M B, Narchet, F M, & Meissner, C A. Investigating the effects of presenting false evidence on true and false confession rates. In: American Psychology-Law Society Annual Conference. 2005.

### Smalarz et al., 2011 {unpublished data only}

- Smalarz, L, Greathouse, S, Madon, S, & Guyll, M. Differential effects of plausible versus implausible false evidence on confession rates. In: American Psychology-Law Society Annual Conference. 2011.

### Swanner et al., 2010 {published data only}

- Swanner, J K, Beike, D R, & Cole, A T. Snitching, lies and computer crashes: An experimental investigation of secondary confessions. Law and Human Behavior 2010;34(1):53-65. [DOI: 10.1007/s10979-008-9173-5]

### Villalba, 2014 {published data only}

- Villalba, D K. The effect of rapport building in police interrogations: Can rapport improve the diagnosticity of confession evidence? Unpublished dissertation (Florida International University) 2014.

### Wachi et al., 2018 {published data only}

- Wachi, T, Kuraishi, H, Watanabe, K, Otsuka, Y, Yokota, K, & Lamb, M E. Effects of rapport building on confessions in an experimental paradigm. Psychology, Public Policy, and Law 2018;24(1):36-47. [DOI: 10.1037/law0000152]

### Wilford and Wells, 2018 {published data only}

- Wilford, M M, & Wells, G L. Bluffed by the dealer: Distinguishing false pleas from false confessions. Psychology, Public Policy, and Law 2018;24(2):158-170. [DOI: 10.1037/law0000165]

### Woestehoff, 2016 {published and unpublished data}

- Woestehoff, S. "It's (not) your fault": The influence of blame mitigation versus guilt induction on true and false confessions. Unpublished dissertation (University of Texas at El Paso) 2016.

### Wright, 2013 {published data only}

- Wright, D S. The mediating and moderating factors of fabricated evidence on false confessions, beliefs and memory. Unpublished thesis [University of Warwick] 2013.
