# Supplementary material for: Interview and interrogation methods and their effects on true and false confessions: A systematic review update and extension
Source: Campbell Syst Rev. 2024 Oct 10;20(4):e1441. doi: 10.1002/cl2.1441 (PMC11465838; doi:10.1002/cl2.1441)
Supplement: Supplementary file 3 — Supplementary material 3: CA000277‐SUP‐03‐characteristicsOfExcludedStudies.html Characteristics of excluded studies. [file CL2-20-e1441-s004.html]

Characteristics of excluded studies


# Supplementary material 3 to: Interview and Interrogation Methods and their Effects on True and False Confessions: An Update and Extension

Catlin M, Wilson D, Redlich AD, Bettens T, Meissner C, Bhatt S, Brandon S
  
https://doi.org/10.1002/14651858.CA000277

The material in this section has been supplied by the author(s) for publication under a Licence for Publication and the author(s) are solely responsible for the material. Cochrane has reviewed this material, but Cochrane has not copyedited, formatted or proofread. Cochrane accordingly gives no representations or warranties of any kind in relation to, and accepts no liability for any reliance on or use of, such material.

Back to top

# Characteristics of excluded studies

## Table of contents

- Studies ordered by Study ID
  - Billings et al., 2007
  - Dunbar et al., 2014
  - Dunbar et al., 2015
  - Evans et al., 2014
  - Horgan et al., 2012
  - Horselenberg et al., 2003
  - Kebbell et al., 2004
  - Lyon et al., 2014
  - McMillen, 2018
  - Nash & Wade, 2009
  - Newring and O'Donohue, 2008
  - Rush et al., 2017
  - Salvati & Houck, 2019
  - Schrantz, 2014
  - Skerritt-Perta, 2017
  - Zapata, 2018
- References to studies

## Studies ordered by Study ID

| Study | Reason for exclusion |
| --- | --- |
| Billings et al., 2007 | The children (participants) witnessed the act of wrongdoing, but did not participate in the act themselves. Thus, according to our eligibility criteria, any confess they could provide would be a secondary confession as opposed to a primary confession. |
| Dunbar et al., 2014 | Did not measure confession as an outcome |
| Dunbar et al., 2015 | Study was on deception detection, not confessions. |
| Evans et al., 2014 | Participants were not asked to sign a confession, like in other studies. Instead, it was coded whether participants made particular admissions of guilty knowledge. |
| Horgan et al., 2012 | Could not isolate the effect of minization and maximization because of the design. |
| Horselenberg et al., 2003 | Did not manipulate interrogation approach. |
| Kebbell et al., 2004 | Ground truth could not be established. |
| Lyon et al., 2014 | The children being interviewed were not suspects, but witnesses/victims of an act of wrongdoing. |
| McMillen, 2018 | McMillen manipulated the type of false evidence, but did not include a contrast group with another interrogation approach. |
| Nash & Wade, 2009 | Manipulated the type of false evidence, but did not include any eligible contrast group. |
| Newring and O'Donohue, 2008 | The interrogation approach was manipulated within-subjects. Our comparison, therefore, would have been between Step 1 and Step 5 of the interrogation with no statistical method for accounting for confessions that occurred in Steps 2-4 or the cumulative interrogation effect (as opposed to the direct effect of the interrogation approach). |
| Rush et al., 2017 | The children being interviewed were not suspects, but witnesses/victims of an act of wrongdoing. |
| Salvati & Houck, 2019 | This study was conducted online so that participants read about an act of wrongdoing, but were not directly involved. Furtherore, they read an interrogation script instead of engaging with an interrogation as in all the other studies. |
| Schrantz, 2014 | Did not manipulate interrogation approach. |
| Skerritt-Perta, 2017 | Study manipulated the type of false evidence, but did not include another contrast. |
| Zapata, 2018 | Does not manipulate interrogation approach. Instead is focused on a framing effect. |

## References to studies

### Billings et al., 2007 {published data only}

- Billings, F J, Taylor, T, Burns, J, Corey, D L, Garven, S, & Wood, J M. Can reinforcement induce children to falsely incriminate themselves? Law & Human Behavior 2007;31:125-139. [DOI: 10.1007/s10979-006-9049-5]

### Dunbar et al., 2014 {published data only}

- Dunbar, N E, Jensen, M L, Tower, D C, & Burgoon, J K. Synchronization of nonverbal behaviors in detecting mediated and non-mediated deception. Journal of Nonverbal Behavior 2014;38(3):355-376. [DOI: 10.1007/s10919-014-0179-z]

### Dunbar et al., 2015 {published data only}

- Dunbar, N E, Jensen, M L, Burgoon, J K, Kelley, K M, Harrison, K J, Adame, B J, & Bernard, D R. Effects of veracity, modality, and sanctioning on credibility assessment during mediated and unmediated interviews. Communication Research 2015;42(5):649-674. [DOI: 10.1177/0093650213480175]

### Evans et al., 2014 {published data only}

- Evans, J R, Houston, K A, Meissner, C A, Ross, A B, Labianca, J R, Woestehoff, S A, & Kleinman, S M. An empirical evaluation of intelligence-gathering interrogation techniques from the United States army field manual. Applied Cognitive Psychology  2014;28(6):867-875. [DOI: 10.1002/acp.3065]

### Horgan et al., 2012 {published data only}

- Horgan, A J, Russano, M B, Meissner, C A, & Evans, J R. Minimization and maximization techniques: Assessing the perceived consequences of confessing and confession diagnosticity. Psychology, Crime & Law 2012;18(1):65-78. [DOI: 10.1080/1068316X.2011.561801]

### Horselenberg et al., 2003 {published data only}

- Horselenberg, R, Merckelbach, H, & Josephs, S. Individual differences and false confessions: A conceptual replication of Kassin and Kiechel (1996). Psychology, Crime & Law 2003;9(1):1-8. [DOI: 10.1080/10683160308141]

### Kebbell et al., 2004 {published data only}

- Kebbell, M, Hurren, E, & Mazerolle, P. An investigation into the effective and ethical interviewing of suspected sex offenders. Crime and Misconduct Commission and Criminology Research Council Final Report 2004.

### Lyon et al., 2014 {published data only}

- Lyon, T D, Wandrey, L, Ahern, E, Licht, R, Sim, M P Y, & Quas, J A. Eliciting maltreated and nonmaltreated children's transgression disclosures: Narrative practice rapport building and a putative confession. Child Development 2014;85(4):1756-1769. [DOI: 10.1111/cdev.12223]

### McMillen, 2018 {published data only}

- McMillen, K. Imagination or proof: The use of imagery and false evidence in eliciting internalized false confessions. Unpublished thesis [John Jay College of Criminal Justice] 2018.

### Nash & Wade, 2009 {published data only}

- Nash, R A, & Wade, K A. Innocent but proven guilty: Eliciting internalized false confessions using doctored-video evidence. Applied Cognitive Psychology 2009;23(5):624-637. [DOI: 10.1002/acp.1500]

### Newring and O'Donohue, 2008 {published data only}

- Newring, K A B, & O'Donohue, W. False confessions and influenced witnesses. Applied Psychology in Criminal Justice 2008;4(1):81-107.

### Rush et al., 2017 {published data only}

- Rush, E B, Stolzenberg, S N, Quas, J A, & Lyon, T D. The effects of the putative confession and parent suggestion on children's disclosure of a minor transgression. Legal and Criminological Psychology 2017;22(1):60-73. [DOI: 10.1111/lcrp.12086]

### Salvati & Houck, 2019 {published data only}

- Salvati, J M, & Houck, S C. Examining the causes and consequences of confession-eliciting tactics during interrogation. Journal of Applied Security Research 2019;14(3):241-256. [DOI: 10.1080/19361610.2019.1621508]

### Schrantz, 2014 {published data only}

- Schrantz, K. Personality and situational correlates of false confessions. Unpublished thesis [University of Central Oklahoma] 2014.

### Skerritt-Perta, 2017 {published data only}

- Skerritt-Perta, A S. Comparison of the effects of two types of false evidence on rates of false confession in a Reid technique-style interrogation paradigm. Unpublished thesis [California State University].

### Zapata, 2018 {published data only}

- Zapata, C. Effects of message-framing and psychopathic personality traits on eliciting false confessions in university students. Unpublished dissertation [ProQuest Number: 10824455] 2018.
