# Supplementary material for: Interview and interrogation methods and their effects on true and false confessions: A systematic review update and extension
Source: Campbell Syst Rev. 2024 Oct 10;20(4):e1441. doi: 10.1002/cl2.1441 (PMC11465838; doi:10.1002/cl2.1441)
Supplement: Supplementary file 4 — Supplementary material 5: CA000277‐SUP‐05‐other.html Individuals Contacted. [file CL2-20-e1441-s002.html]

Individuals Contacted


# Supplementary material 5 to: Interview and Interrogation Methods and their Effects on True and False Confessions: An Update and Extension

Catlin M, Wilson D, Redlich AD, Bettens T, Meissner C, Bhatt S, Brandon S
  
https://doi.org/10.1002/14651858.CA000277

The material in this section has been supplied by the author(s) for publication under a Licence for Publication and the author(s) are solely responsible for the material. Cochrane has reviewed this material, but Cochrane has not copyedited, formatted or proofread. Cochrane accordingly gives no representations or warranties of any kind in relation to, and accepts no liability for any reliance on or use of, such material.

Back to top

# Individuals Contacted

|  |  |
| --- | --- |
| **Name** | **Affliation** |
| Iris Blandón-Gitlin | California State University, Fullerton, FL, USA |
| Randy Borum | University of South Florida, FL, USA |
| Joseph Buckley | John Reid and Associates, IL, USA |
| Stephanie Cardenas | Williams College, MA, USA |
| Julie Cherryman | University of Portsmouth, United Kingdom |
| Mark Costanzo | Claremont Graduate School, CA, USA |
| Brian Cutler | Ontario Tech University, Canada |
| David Dixon | University of New South Wales, Australia |
| Steven Drizin | Northwestern School of Law, IL, USA |
| Jacqueline Evans | Florida International University, FL, USA |
| Ronald Fisher | Florida International University, FL, USA |
| Par Anders Granhag | Goteborg University, Sweden |
| Max Guyll | Iowa State University, IA, USA |
| Maria Hartwig | John Jay College of Criminal Justice, NY, USA |
| Lorraine Hope | University of Portsmouth, United Kingdom |
| Saul Kassin | John Jay College of Criminal Justice, NY, USA |
| Mark Kebbell | Griffith University |
| Christopher Kelly | St. Joseph's University, PA, USA |
| Steven Kleinman | MacDill Air Force Base, FL, USA |
| Gunther Kohnken | University of Kiel, Germany |
| Jeff Kukucka | Towson University, MD, USA |
| Michael Lamb | University of Cambridge, United Kingdom |
| Amy Leach | Ontario Tech University, Canada |
| Richard Leo | University of San Francisco School of Law, CA, USA |
| Stephanie Madon | Iowa State University, IA, USA |
| Samantha Mann | University of Portsmouth, United Kingdom |
| Jaume Masip | University of Salamanca, Spain |
| Rebecca Milne | University of Portsmouth, United Kingdom |
| Amelia Mindthoff | Iowa State University, IA, USA |
| Fadia Narchet | University of New Haven, CT, USA |
| Christopher Normile | Allegheny College, PA, USA |
| Jennifer Perillo | Indiana University of Pennsylvania, PA, USA |
| Melissa Russano | Roger Williams University, RI, USA |
| Kyle Scherr | Central Michigan University, MI, USA |
| Laura Smalarz | Arizona State University, AZ, USA |
| Brent Snook | Memorial University of Newfoundland, Canada |
| Leif Stromwall | University of Gothenburg, Sweden |
| Paul Taylor | Lancaster University, United Kingdom |
| Aldert Vrij | University of Portsmouth, United Kingdom |

[Enter text here]
